# Nanocomposite hydrogels for biomedical applications

**DOI:** 10.1002/btm2.10315

**Published:** 2022-04-09

**Authors:** Shanghui Huang, Xiangqian Hong, Mingyi Zhao, Nanbo Liu, Huiling Liu, Jun Zhao, Longquan Shao, Wei Xue, Han Zhang, Ping Zhu, Rui Guo

**Affiliations:** ^1^ Key Laboratory of Biomaterials of Guangdong Higher Education Institutes, Guangdong Provincial Engineering and Technological Research Centre for Drug Carrier Development, Department of Biomedical Engineering Jinan University Guangzhou China; ^2^ Institute of Microscale Optoelectronics, Collaborative Innovation Centre for Optoelectronic Science & Technology, International Collaborative Laboratory of 2D Materials for Optoelectronics Science and Technology of Ministry of Education, Key Laboratory of Optoelectronic Devices and Systems of Ministry of Education and Guangdong Province, Shenzhen Key Laboratory of Micro‐Nano Photonic Information Technology, Guangdong Laboratory of Artificial Intelligence and Digital Economy (SZ) College of Physics and Optoelectronic Engineering, Shenzhen University Shenzhen China; ^3^ Shenzhen Eye Hospital, Shenzhen Eye Institute, Shenzhen Eye Hospital affiliated to Jinan University, School of Optometry, Shenzhen University Shenzhen China; ^4^ Guangdong Cardiovascular Institute, Guangdong Provincial People's Hospital, Guangdong Academy of Medical Sciences Guangzhou China; ^5^ Department of Ophthalmology Shenzhen People's Hospital (The Second Clinical Medical College, Jinan University; The First Affiliated Hospital, Southern University of Science and Technology) Shenzhen China; ^6^ Stomatological Hospital, Southern Medical University Guangzhou China

**Keywords:** bioimaging, biosensing, cancer treatment, drug and cell delivery, nanocomposite hydrogels, tissue regeneration

## Abstract

Nanomaterials' unique structures at the nanometer level determine their incredible functions, and based on this, they can be widely used in the field of nanomedicine. However, nanomaterials do possess disadvantages that cannot be ignored, such as burst release, rapid elimination, and poor bioadhesion. Hydrogels are scaffolds with three‐dimensional structures, and they exhibit good biocompatibility and drug release capacity. Hydrogels are also associated with disadvantages for biomedical applications such as poor anti‐tumor capability, weak bioimaging capability, limited responsiveness, and so on. Incorporating nanomaterials into the 3D hydrogel network through physical or chemical covalent action may be an effective method to avoid their disadvantages. In nanocomposite hydrogel systems, multifunctional nanomaterials often work as the function core, giving the hydrogels a variety of properties (such as photo‐thermal conversion, magnetothermal conversion, conductivity, targeting tumor, etc.). While, hydrogels can effectively improve the retention effect of nanomaterials and make the nanoparticles have good plasticity to adapt to various biomedical applications (such as various biosensors). Nanocomposite hydrogel systems have broad application prospects in biomedicine. In this review, we comprehensively summarize and discuss the most recent advances of nanomaterials composite hydrogels in biomedicine, including drug and cell delivery, cancer treatment, tissue regeneration, biosensing, and bioimaging, and we also briefly discussed the current situation of their commoditization in biomedicine.

## 1 INTRODUCTION

Nanomaterials can be divided into zero‐dimensional materials (0DM), one‐dimensional materials (1DM), and two‐dimensional materials (2DM) according to their size, length‐diameter ratio, and diameter‐thickness ratio.^1^ Nanomaterials exhibit huge application potentials in various fields based on their nano size, diverse structures, outstanding mechanical characteristics, special electrical conductivity, thermal conductivity, light responsiveness, and environmental friendliness.^2^, ^3^, ^4^, ^5^, ^6^, ^7^ Currently, a large number of studies have been conducted to examine nanomaterials in catalysis,^8^ energy,^9^, ^10^ semiconductors,^4^ supercapacitors,^11^ fiber lasers,^12^, ^13^ photonic devices,^13^ and biomedicine.^14^, ^15^ In biomedicine, some nanomaterials have attracted the attention of researchers due to their good biocompatibility and excellent mechanical, thermal, electrical, and optical properties.^1^, ^16^, ^17^ Nanomaterials can be cleared by the mononuclear phagocytic system (MPS).^18^ However, nanomaterials do possess some prominent shortcomings, including burst release, rapid elimination, poor biological adhesion, and irreversible deformation.^19^ Because of the small size, nanomaterials will have an initial burst release (reaching the release peak in the initial short term). The high concentration during burst release may cause toxicity, and the concentration will decrease rapidly after that, resulting in limited therapeutic effect. Due to the small size of nanoparticles, they may be quickly eliminated by the body's reticuloendothelial system.^19^ And poor bioadhesion may lead to the off‐target of nanomaterials. More importantly, nanomaterials alone can be used for in vivo injection but cannot be used alone for in vitro applications (such as various biosensors). Therefore, it is necessary to combine other materials, which cannot only improve the above shortcomings of nanomaterials but also expand their biomedical applications in vitro.^20^, ^21^, ^22^, ^23^

Among many bioscaffold materials, hydrogels are one of the most extensively studied.^24^ Hydrogels are a type of soft, bionic material composed of a 3D network structure, and can store a large amount of water.^25^ Hydrogels can meet many requirements in biomedicine, where they can function in drug and cell delivery, as tissue repairing scaffolds, in replacement of extracellular matrix, and imitation of artificial organs.^25^, ^26^ The gelation process of hydrogels primarily occurs due to supramolecular interactions or chemical covalent bonds.^27^ In general, polymers can be modified by introducing new functional groups or polymer components to obtain injectable or self‐repairing hydrogels.^18^ Hydrogels are scaffolds with good biocompatibility and drug release capacity. They have good plasticity to adapt to various biomedical applications.^24^ While hydrogels are also associated with disadvantages for biomedical applications such as poor anti‐tumor capability, weak bioimaging capability, limited responsiveness, and so on. Moreover, hydrogels usually have weak mechanical properties and cannot meet the needs of some biomedical applications (such as cartilage/bone repair).^28^ It is necessary to combine hydrogels with other materials to improve the above shortcomings of hydrogels.

**FIGURE 1:**
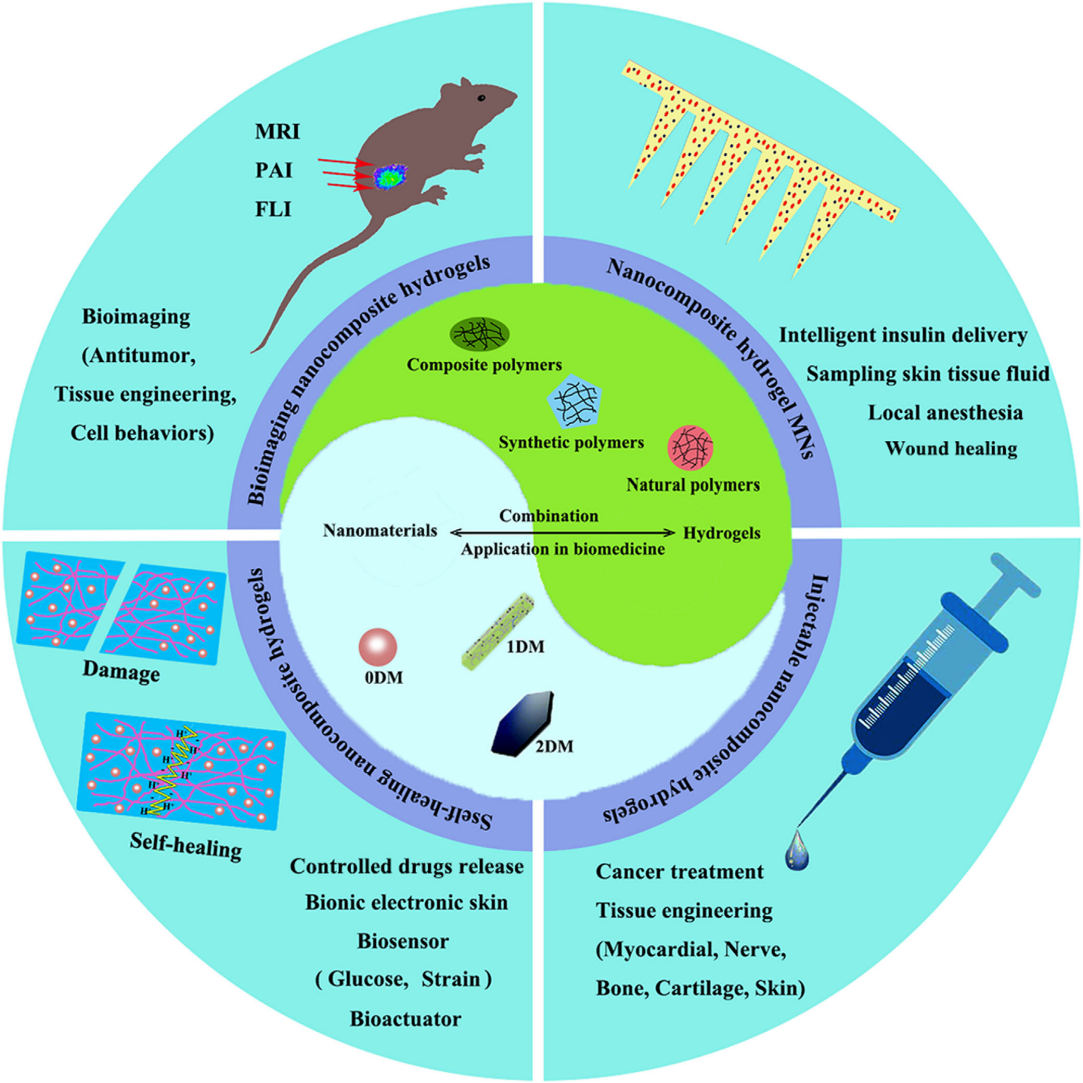
Schematic diagram of the main content of this review. Nanomaterials can be divided into zero‐dimensional materials (0DM), one‐dimensional materials (1DM), and two‐dimensional materials (2DM) according to their size, length‐diameter ratio, and diameter‐thickness ratio. Based on the polymer composition, hydrogels can be distributed into the natural polymer, synthetic polymer, and composite polymer hydrogels. According to the macroscopic phenotype of the composite systems, nanomaterials and hydrogels mainly combined into four composite systems, including nanocomposite hydrogel microneedles (MNs), injectable nanocomposite hydrogels, self‐healing nanocomposite hydrogels and bioimaging nanocomposite hydrogels. We reviewed the recent advances and future challenges of the composite systems, which almost involve all areas of biomedicine, including drug and cell delivery, cancer treatment, tissue regeneration, biosensing, and bioimaging

Therefore, nanomaterials composite hydrogels will be a promising method for biomedical application. Incorporating nanomaterials into the 3D hydrogel network through physical or chemical covalent action can effectively avoid their disadvantages. In nanocomposite hydrogel systems, multifunctional nanomaterials often work as the function cores, giving the composite systems a variety of properties (such as photothermal conversion, magnetothermal conversion, conductivity, targeting tumor, etc.).^29^, ^30^, ^31^ Hydrogels can effectively improve the retention effect of nanomaterials and make the composite systems have good plasticity to adapt to various biomedical applications.^18^, ^32^ According to the application requirements, specific nanomaterial composite hydrogel systems can be designed and applied to various fields of biomedicine (e.g., nanomaterials composite hydrogels for preparing insulin microneedle [MN] patches).^33^ Nanomaterial composite hydrogel systems have broad application prospects in biomedicine. Herein, we summarized the composition and classification of nanomaterials and hydrogels, mainly focused on the most recent advances of nanomaterials composite hydrogels in biomedicine (Figure 1). The review summarized almost all areas of biomedicine, including drug and cell delivery, cancer treatment, tissue regeneration, biosensing, and bioimaging. We also briefly discussed the current situation of their commoditization in biomedicine.

## 2 RECENT ADVANCES IN NANOMATERIALS

**FIGURE 2:**
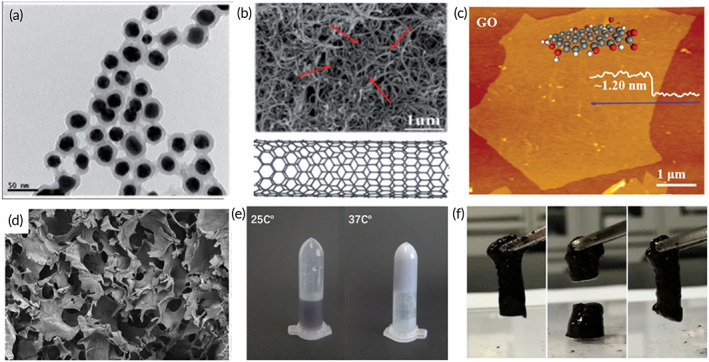
Representative samples of nanomaterials and hydrogels. (a) TEM image of Au NPs@SiO_2_ (0DM). *Source*: Adapted with permission from ref 59. Copyright 2020, the Royal Society of Chemistry. (b) SEM image and schematic diagram of CNTs (1DM). *Source*: Adapted with permission from ref 60 Copyright 2020, the Royal Society of Chemistry. (c) Atomic force microscopy (AFM) image of single‐layer GO flake (2DM). *Source*: Adapted with permission from ref 61. Copyright 2020, WILEY‐VCH. (d) SEM image of Chitosan/β‐glycerophosphate sodium hydrogel. *Source*: Adapted with permission from ref 52. Copyright 2020, Elsevier. (e) An image of injectable poly(caprolactone)‐poly(ethylene glycol)‐poly (caprolactone) thermosensitive hydrogel. *Source*: Adapted with permission from ref 54. Copyright 2018, American Chemical Society. (f) Photographs showing the self‐healing ability of hydrogel prepared by quaternized chitosan and oxidized dextran. *Source*: Adapted with permission from ref 57. Copyright 2020, Elsevier

In nano structures, 0DM can be divided into solid, interpore, hollow, and so on; 1DM can be divided into linear structure, rod, hollow fiber, and so on; 2DM can be divided into single layer and multiple layers (Table 1). Nanomaterials possess many excellent functions due to their nanoscale size and rich structures. The 3D sizes of 0DM do not differ too significantly (length: width: height ≈ 1) (Figure 2a).^1^ Over the past few decades, various 0DM have been successfully prepared, such as gold nanoparticles (Au NPs),^34^ mesoporous silica nanoparticles (MSNs),^34^ silver nanoparticles (Ag NPs),^36^ and titanium dioxide (TiO_2_) nanoparticles.^37^ Au NPs, as a kind of precious metal nanoparticles, have rich properties due to their nanostructure: good biosafety, phototherapeutic properties, surface modification, bioimaging, and so on. For example, Au NPs have been used as alternative nanoprobes for live cell imaging by dark field microscopy (DFM).^38^

**TABLE 1:** Representative nanomaterials and hydrogels

Nano and gel	Class	Materials	Properties	Behaviors	Shortcomings	References
Nanomaterials	0DM	Au NPs	Nanograde solid gold particles	Photothermal properties, biological imaging, electrical conductivity	Poor adhesion, easy to be removed, particle precipitation	34
		MSNs NPs	Nano‐mesoporous structure	Drug loading capacity	Poor adhesion, easy to be removed, particle precipitation	35
		Ag NPs	Nanograde solid silver particles	Antimicrobial ability, anti‐tumor	Poor adhesion, easy to be removed, particle precipitation	36
		TiO_2_ NPs	Crystalline/amorphous nuclear shell structure	Photothermal properties, photodynamic properties	Poor adhesion, easy to be removed, particle precipitation	37
	1DM	CNCs	Cellulose nano‐rods	High surface energy, mechanical enhancement performance	Poor adhesion, easy to be removed, particle precipitation	38
		Au NWs	Linear nanogold fiber	High aspect ratio, unique electrical, optical, and magnetism	Poor adhesion, easy to be removed, particle precipitation	35
		CNTs	Hollow fibrous structure with nano radial size	Good electrical and chemical properties, high modulus, and high strength	Poor adhesion, particle precipitation	3
	2DM	GO NSs	Two‐dimensional graphene oxide nanosheets, single or multiple layers	Photothermal properties, antibacterial activity, and drug loading properties	Poor adhesion, easy to be removed, particle precipitation	39, 40
		BP NSs	Two‐dimensional black phosphorus nanosheets, single or multiple layers	Photothermal properties, photodynamic properties, drug‐loading properties, and biological imaging	Poor adhesion, easy to be oxidative degradation, particle precipitation	41, 42, 43, 44, 45, 46, 47
		MXene NSs	Two‐dimensional transition metal nanosheets, single or multiple layers	High electrical conductivity, photoelectromagnetic characteristics, and mechanical enhancement capability	Poor adhesion, easy to be removed, particle precipitation	48, 49, 50, 51
Hydrogels	Natural polymer hydrogels	Chitosan	Cationic polysaccharide	Antibacterial activity, 3D scaffold, and drug loading capability	Weak mechanical property, required in combination with drugs or cells	52
		Dextran	Nonionic polysaccharide	High water content, 3D scaffold, drug loading capability	Weak mechanical property, required in combination with drugs or cells	52
		Cellulose	Nonionic polysaccharide	High water content, 3D scaffold, drug loading capability	Required in combination with drugs or cells	45
		Alginate	Ca^2+^ Sensitive natural polysaccharide	High water content, 3D scaffold, drug loading capability	Required in combination with drugs or cells	53
	Synthetic polymer hydrogels	Poly(caprolactone)–poly(ethyleneglycol)–poly(caprolactone)	Temperature‐sensitive synthetic polymers	Injectability, 3D scaffold, drug loading capability	Required in combination with drugs or cells	54
		Poly(acrylamide‐*co*‐maleic anhydride) (P (AM‐*co*‐MAH))	Rich in hydrogen bonds	Self‐healing, drug loading capability, high mechanical strength, stretching ability, 3D scaffold	Required in combination with drugs or cells	55
		Polymethyl vinyl ether‐salt‐maleic acid	hydrophilic group (—COOH)	Large swelling capacity, 3D scaffold, drug loading capability	Required in combination with drugs	56
	Composite polymer hydrogels	Methacrylate‐modified gelatin and hyaluronic acid grafting dopamine	Photopolymerizable double bond, hydrophilic group (—OH, —NH_2_, —COOH)	Injectable, 3D scaffold, drug loading capability, adhesion	Required in combination with drugs	40
		Quaternized chitosan and dextran	Dynamic chemical bond (Schiff base bond)	Self‐healing, 3D scaffold, drug loading capability	Weak mechanical property, required in combination with drugs	57
		3‐Aminophenylboronic acid, aniline, and polyvinyl alcohol	Ultramolecular assembly of hydrogels, dynamic bonds (hydrogen bonding and π–π stacking)	Self‐healing, 3D scaffold, drug loading capability, electric conduction	Required in combination with nanoparticles to expand or enhance performance	58

1DM refers to materials possessing a relatively large length‐to‐diameter ratio (Figure 2b), such as nanocrystalline cellulose (CNCs),^62^ gold nanowires (Au NWs),^63^ carbon nanotubes (CNTs),^3^ and so on. 1DM often exhibits a high degree of anisotropy and based on this, it simultaneously exhibits many excellent properties such as extremely high tensile strength and specific responding to temperature and humidity.^64^, ^65^ For example, CNTs, as a new carbon‐based nanomaterials containing hollow fibrous structure, have properties of high tensile strength, gas adsorption, high conductivity, and thermal conductivity. Thus, it is commonly used for mechanical and conductive enhancement of macroscopic materials, commonly used in the field of biosensing.^66^

2DM refer to materials that possess a relatively large diameter–thickness ratio (Figure 2C) such as graphene,^61^, ^67^, ^68^ reduced graphene oxide (rGO),^69^ graphene oxide (GO),^39^, ^40^ phosphorene,^44^, ^70^, ^71^ MXene,^48^, ^49^, ^50^, ^51^ perovskite layered molybdenum disulfide (MoS_2_),^72^ SnS,^73^, ^74^, ^75^ and mono‐elemental 2D materials such as black phosphorus (BP) nanosheets,^41^, ^42^, ^43^, ^44^, ^45^, ^46^, ^47^ tellurene,^76^, ^77^, ^78^ antimonene,^79^, ^80^, ^81^ borophene,^14^ bismuthene,^82^, ^83^, ^84^, ^85^ 2D selenium,^86^, ^87^ 2D metal,^88^ and so on. Regarding the applicability, 2DM possesses unique optical, mechanical, electrical, and sensing properties based on their sheet and layer structures, and they can therefore be extensively used in biomedicine.^17^, ^44^, ^78^

Research examining 2DM began at a much later date than that of 0DM and 1DM. The research wave of 2DM began in 2004 when scientists successfully prepared the first graphene nanosheets.^42^, ^89^ As a typical 2D material, graphene has exhibited considerable research achievements over the past 10 years.^69^, ^90^ Graphene is widely used in biomedical fields, such as high‐sensitivity biosensors, drug delivery systems, cell imaging, tissue engineering, photothermal therapy (PTT), and near‐infrared fluorescence imaging.^91^ BP is also a representative 2DM in recent years. BP can be used as a potential bioimaging agent by virtue of its inherent fluorescence (FL) and its photoacoustic (PA) characteristics.^41^, ^92^ Based on its excellent physical characteristics (such as NIR light absorption,^47^, ^93^ large extinction coefficient, great photothermal conversion efficiency, and ability to generate reactive oxygen species [ROS]), BP exerts excellent anti‐cancer effects (PTT^43^, ^94^ and photodynamic therapy [PDT]^45^). BP exhibits a fold layered structure with a large surface area, providing an excellent platform for transporting medicine.^41^, ^42^, ^47^ Additionally, BP exhibits controllable surface chemical modification ability, allowing it to greatly enrich its functional responsiveness and improving its stability.^95^, ^96^

## 3 RECENT ADVANCES IN HYDROGELS

According to the polymer composition, hydrogels can be distributed into the natural polymer, synthetic polymer, and composite polymer hydrogels (Table 1). Natural polymer hydrogels exhibit good biocompatibility and biodegradability, and hence they possess very promising application potentials in biomedicine. For example, researchers have used chitosan and β‐glycerophosphate to prepare an injectable thermosensitive hydrogel (Figure 2d).^52^ Dextran and cellulose, as typical nonionic natural polysaccharide hydrogels, usually need to be combined with cells or nano drugs to meet specific requirements.^45^, ^97^ Sodium alginate is sensitive to Ca^2+^ and has good biocompatibility. It can load cells or nano drugs for human body.^53^ Synthetic components also possess excellent properties and are easy to design and modify, and they also play an indispensable part in the construction of hydrogels. For example, researchers have used poly(caprolactone)‐poly(ethyleneglycol)‐poly(caprolactone) to develop an injectable thermosensitive hydrogel (Figure 2e).^54^ Poly(acrylamide‐*co*‐maleic anhydride) (P(AM‐*co*‐MAH)) hydrogel is self‐healing because of rich hydrogen bonds, and polymethyl vinyl ether‐salt‐maleic acid hydrogel possesses large swelling capacity due to the hydrophilic group (—COOH).^55^, ^56^

For practical applications, hydrogels are often required to simultaneously possess many properties such as good biocompatibility, biodegradability, proper hydrophilic, good mechanical properties, and special functional groups. However, many natural polymers or synthetic polymers cannot meet the above requirements alone. Therefore, it may be good to design multifunctional composites containing multiple natural polymers or synthetic polymers. Mechanical properties are one of the important parameters of hydrogels, and mechanical properties of corresponding hydrogels should be designed according to their uses. For example, the hydrogel used for bone repair has strong mechanical properties matching the bone (Young modulus ≈30–60 kPa),^32^ and the hydrogel used for skin repair has weak mechanical properties matching the skin (Young modulus ≈5–20 kPa).^40^ Hydrogels that possess high mechanical strength can be obtained by improving the degree of cross‐linking.^35^ For example, Huang et al. developed a composite polymer hydrogel with methacrylate‐modified gelatin and hyaluronic acid grafting dopamine. By increasing the crosslinking degree of methacrylate‐modified gelatin, the hydrogel obtained better mechanical properties.^40^ Moreover, researchers have developed a composite polymer hydrogel based on quaternized chitosan and dextran that possesses self‐healing properties (Figure 2f).^57^ The designed composite hydrogel can simultaneously possess the excellent properties of natural or synthetic polymers to achieve synergistic complementarity.^98^ Hydrogels that are able to intelligently respond to environmental changes (such as heat, pH, light, and ultrasound) can be designed by introducing relevant functional groups to achieve in situ gelation and controlled drug release.^99^ For example, groups with double bonds can be grafted onto dextran by transesterification. When exposed to UV light, double bonds can be polymerized to form photo‐sensitive hydrogels.^100^ It is also possible to design self‐healing hydrogels by introducing dynamic cross‐linking.^98^ For example, the hydrogel that based on 3‐aminophenylboronic acid, aniline, and polyvinyl alcohol, has strong self‐healing ability because of the dynamic cross‐linking (hydrogen bonding and π–π stacking).^101^

## 4 RECENT ADVANCES OF NANOCOMPOSITE HYDROGELS IN BIOMEDICAL ENGINEERING

Nanomaterials have successfully demonstrated their huge application potential in nanomedicine by virtue of their various distinctive physical and chemical characteristics.^15^, ^42^, ^45^ For example, Au NPs have good catalytic activity, photothermal conversion performance, biological imaging performance, and electrical conductivity.^38^ However, they do exhibit some shortcomings that inevitably hinder their application effect and practicality (such as burst release, rapid removal, poor biological adhesion, and irreversible deformation).^19^ As 3D scaffolds, hydrogels alone are often associated with limited therapeutic effects, because of their poor anti‐tumor capability, weak bioimaging capability, limited responsiveness, and so on. However, biocompatible hydrogels exhibit excellent encapsulation ability. For example, chitosan hydrogel can be used to be loaded with stem cells and nanoparticles for skin repairing.^35^ As a promising approach, scientists incorporate nanomaterials into the 3D hydrogel network through physical or chemical covalent action to build composite material systems that can effectively avoid their shortcomings. In composite systems, hydrogels can effectively improve the retention effect of nanomaterials and make the composite systems have good plasticity to adapt to various biomedical applications.^18^, ^32^ Multifunctional nanomaterials often work as the function cores, giving the composite systems various properties (such as photothermal conversion, magnetothermal conversion, conductivity, targeting tumor, etc.).^29^, ^30^, ^31^ Nanomaterial composite hydrogel systems exhibit huge application potentials in biomedicine. According to the macroscopic phenotype of the composite systems, nanomaterials and hydrogels mainly combined into four composite systems, including nanocomposite hydrogel MNs, injectable nanocomposite hydrogels, self‐healing nanocomposite hydrogels, and bioimaging nanocomposite hydrogels. We here reviewed the recent advances and future challenges of the composite systems, which almost involve all areas of biomedicine, including drug and cell delivery, cancer treatment, tissue regeneration, biosensing, and bioimaging.

### 4.1 Recent advances of nanocomposite hydrogel microneedle patches

MN technology has the advantages of painlessness, low invasiveness, low infectivity, and it is an ideal transdermal method.^102^ The length of MNs is usually no more than 1 mm, which is much smaller than the traditional metal injection needles (the length of the traditional metal injection needles is not less than 4 mm).^103^ Additionally, the piercing level of MNs is only at the epidermis and does not damage the nerves and capillaries of the dermis. Thus, they can minimize the pain caused by percutaneous administration and do not cause skin injury or bleeding.^104^ According to the solubility properties of the polymer needles, there are two types of polymer MNs, including soluble or insoluble polymer‐needle MNs. Nanocomposite hydrogels can be used to prepare insoluble polymer MNs. As the host materials are hydrogels, such MN patches exhibit good biocompatibility. Nanomaterial composite hydrogel MNs have been widely studied in biomedicine, such as wearable device applications, intelligent insulin release, noninvasive imaging, skin repairing, local anesthesia, and skin interstitial fluid monitoring.^104^, ^105^ In the composite MNs, hydrogel needle tips are generally used to penetrate the skin. Nanomaterials can expand the functions of MN patches, and they can also enhance the mechanical properties of MNs by interacting with hydrogels (hydrogen bonds, hydrophobic forces, and charge interactions).^106^ Studies show that as long as the mechanical strength of the needle tip is not less than 0.045 N/needle, the MNs can pierce the skin.^107^ As long as the MNs can pierce the skin, the MNs can play an effect. The mechanical strength of the needle tips is not necessarily the stronger the better. Under the interaction of nanomaterials and hydrogels, needles piercing the skin are not such difficult. Therefore, this section mainly focuses on the function improvements and the mechanism of nanocomposite hydrogel MNs.

#### 4.1.1 Recent advances of MN patches for intelligent insulin delivery

Providing patients with life‐long exogenous insulin is extremely important for the treatment of diabetes. Nanocomposite hydrogel MNs possess many advantages, such as minimally invasive, function in real‐time, low infection, and possess sustainable drug transport capabilities. Nanoparticles endow the MNs with the ability to sense blood glucose concentration, thus enabling intelligent delivery of insulin.

**FIGURE 3:**
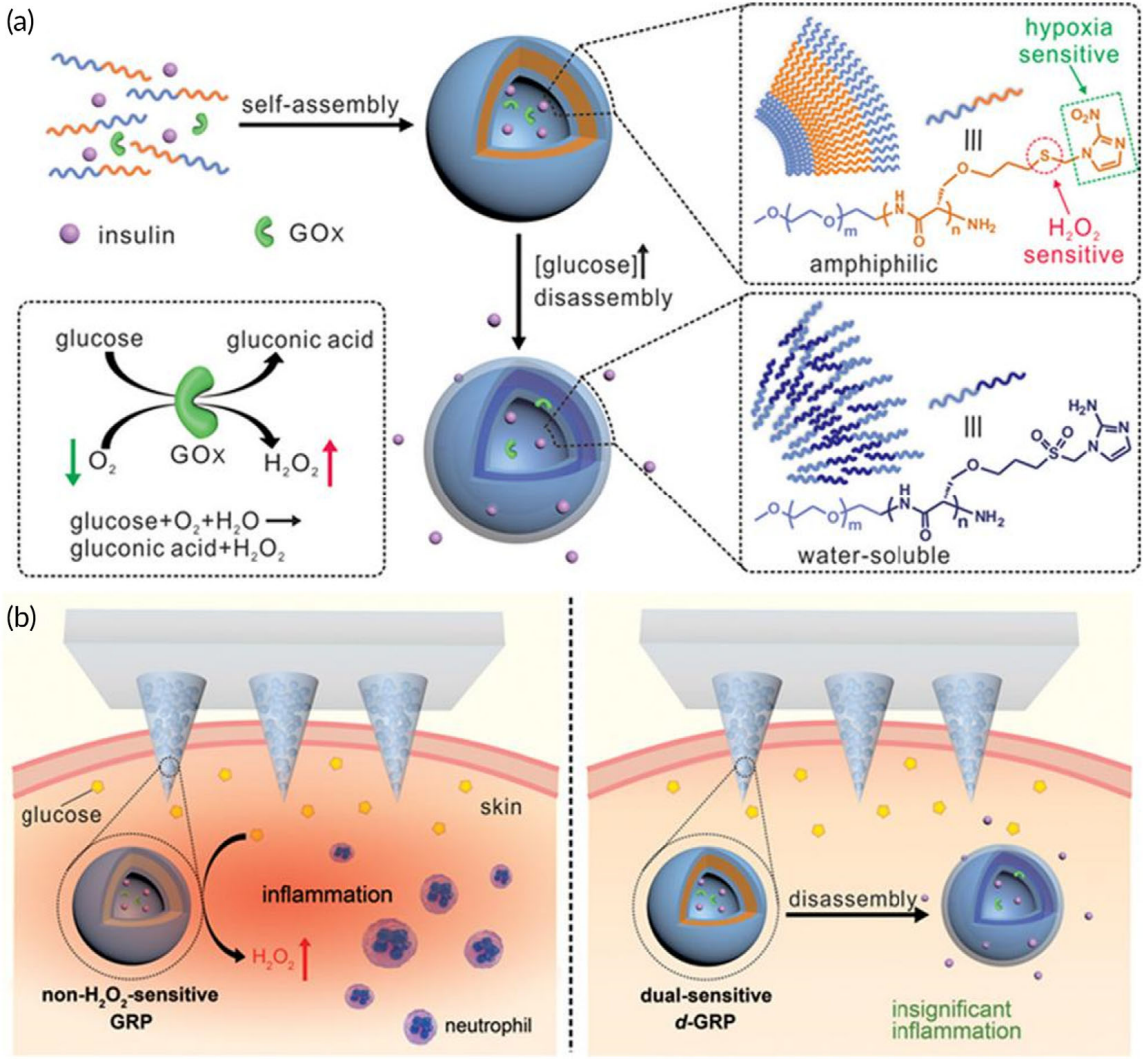
MN patch based on anoxia and H_2_O_2_ bio‐sensitive nanovesicles (d‐GRP) for intelligent delivery of insulin. (a) The synthesis and mechanism of nanovesicles (d‐GRP). (b) MNs patch loaded with non‐H_2_O_2_‐responsive GRP will induce local inflammation. The MN patch loaded with hypoxic and H_2_O_2_ dual‐sensitive d‐GRP can effectively avoid local inflammation. *Source*: Adapted with permission from ref 109. Copyright 2017, American Chemical Society

Yanqi Ye and others have developed a painless MN patch platform. The MNs are composed of cross‐linked hyaluronic acid (HA‐MA) hydrogel and contain a synthetic “glucose signal amplifier” (GSA). This GSA is a self‐assembled polymer nanovesicle that can effectively amplify the blood glucose signal. There are islet B cells attached to the surface of the MN patch, which can regulate blood glucose levels.^33^ This is the first study of MN patches for intelligent regulation of blood glucose balance. Hu et al. integrated H_2_O_2_‐responsive polymer nanovesicles (PVs) with HA‐MA hydrogel MNs to prepare a glucose‐sensitive insulin delivery device. The device could achieve rapid response and painless drug delivery.^108^ Nanomaterials (PVs) were not released from the MNs. The PVs are hollow nanospheres loaded with insulin and glucose oxidase (GOx) inside. When MNs patches pierce into the skin, large amounts of glucose in the body fluid diffuse into the PVs through MNs. Glucose produces a large amount of H_2_O_2_ under the action of GOx, which causes the disintegration of PVs, resulting in the release of insulin from PVs. The released insulin spreads into the skin through body fluids to exert a therapeutic effect. However, the residual H_2_O_2_ within this device is likely to cause local inflammation. Therefore, Yu et al. synthesized hypoxic and H_2_O_2_ dual‐sensitive polymer nanovesicles (d‐GRP) and integrated them with HA‐MA hydrogel MNs to develop a glucose‐responsive insulin transport platform (Figure 3).^109^ Compared to previous devices, this device can eliminate the adverse effects of H_2_O_2_ and reduce inflammation. Moreover, Zhang et al. synthesized a nanocomposite micelle (NC) with dual H_2_O_2_ and pH responses and integrated it with cross‐linked PVA hydrogel MNs to develop a glucose‐sensitive insulin transport platform.^110^ This device can also respond rapidly to blood glucose and can eliminate H_2_O_2_, thus reducing inflammation. Additionally, MNs can combine the dynamic monitoring of blood glucose with intelligent drug delivery. Lee et al. combined a multimodal glucose sensor with MN patches to develop a wearable device that could dynamically monitor glucose and facilitate drug delivery. The MN patch consists of cross‐linked HA hydrogel and temperature‐responsive phase change nanoparticles and could deliver the drug as needed.^111^ Drug‐loaded temperature phase change nanoparticles (PCN) are composed of phase change materials, and drugs (metformin or chloropropamide) are embedded in the matrix. PCN NPs melt above the skin temperature (30°C) to release drugs. MN patch and human sweat detection sensor form an intelligent insulin release device. When the sensor detects abnormal human blood glucose, it will heat the MN patch, resulting in the melting of PCN NPs to release drugs on demand.

It is showed that MNs composed of hydrogels and nanomaterials possess considerable prospects for the intelligent delivery of insulin. Nanomaterials cannot only enhance the mechanism of MNs but also work as blood glucose sensors. Hydrogels have good plasticity and can be used as the main materials of MNs. MNs pierce the skin to form microchannels and deliver the drug to the subcutaneous through the diffusion of the gel needle tips. However, there are still many problems related to clinical applications. For example, the drug loading capacity of MNs is limited. MNs can already control the blood glucose stability of mice. However, when applied to humans, the dosage is far from sufficient, which is the main shortcoming that hinders the large‐scale application of MNs.

#### 4.1.2 Recent advances of MN patches for sampling interstitial fluid

**FIGURE 4:**
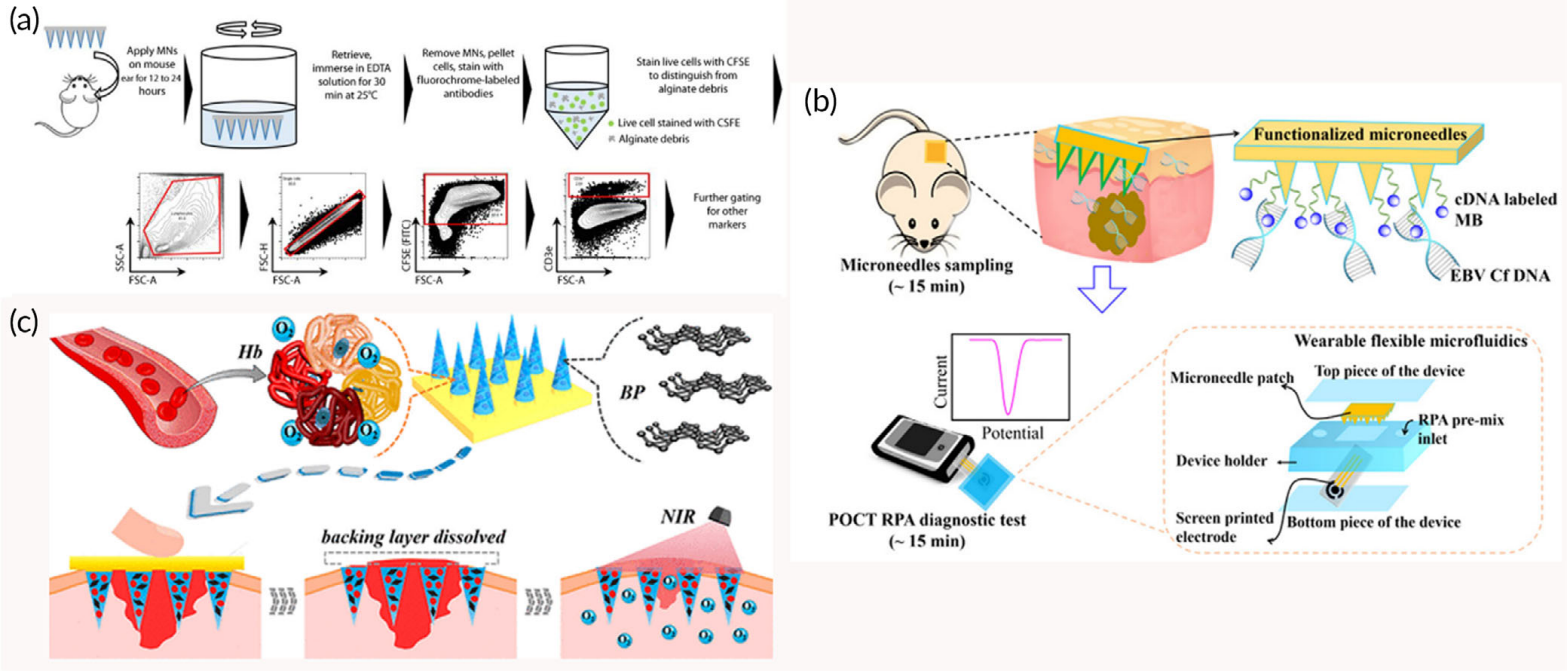
(a) Cell sampling microneedles could be used for analyzing cellular and humoral immune reactions in series. *Source*: Adapted with permission from ref 104. Copyright 2018, American Association for the Advancement of Science. (b) Principles of microneedle patches and electrochemical microfluidic systems. *Source*: Adapted with permission from ref 56. Copyright 2019, American Chemical Society. (c) Separable MNs loaded with black phosphorus (BP) and oxygen carrier for promoting wound healing. *Source*: Adapted with permission from ref 112. Copyright 2020, American Chemical Society

Traditional methods of monitoring the immune system are typically based on blood, saliva, and urine sampling. In contrast, many pivotal immunological cells prefer to gather in surrounding organs such as the skin, intestines, and other mucous membranes. Traditional immune analysis methods cannot analyze these cells. Therefore, Anasuya Mandal and others developed a MN‐based platform that can be used to jointly sample interstitial fluid (ISF) and living cells from the skin (Figure 4a).^104^ The alginate hydrogel was coated onto the solid MNs as the sampling layer, and the nanoparticles carrying the specific antigen and adjuvant were incorporated into the sampling layer to form a stimulating sampling MNs patch. The hydrogel sampling layer absorbs tissue fluid and swells when applied to the skin, forming a porous matrix conducive to leukocyte infiltration, thus enabling a minimally invasive sampling of subcutaneous cells. The specific antigen and adjuvant molecules are encapsulated in nanoparticles, and then the nanomaterials are embedded in the hydrogel coating. The specific antigen and adjuvant molecules encapsulated in nanoparticles can enrich and enhance the proportion of antigen specific lymphocytes in the sampled cell population. Finally, the cell phenotype and function were analyzed in vitro. Information from skin or other mucosal tissues that could only be obtained through invasive biopsy in the past, can now be obtained using MN platform by minimally invasive means. Nasopharyngeal carcinoma is almost asymptomatic in the early stage. However, 80% of the patients presented with local advanced or distant metastasis at a definitive diagnosis. The pathogenesis of the disease is often associated with the Epstein–Barr virus (EBV) cell‐free DNA, a DNA fragment circulating in plasma noncellular components. For these patients, monitoring the amount of EBV‐DNA is extremely important for assessing treatment outcomes and monitoring the course of this disease. Yang et al. introduced an MN patch that can easily capture EBV‐DNA from ISF (Figure 4b).^56^ In this system, the MNs and the electrochemical microfluidics are combined into a wearable device that allows for a real‐time quantitative assay of EBV‐DNA. The MNs were prepared with polymethyl vinyl ether‐salt‐maleic acid (PMVE/MA) hydrogel and gold nanowires (AuNWs). PMVE/MA hydrogel after swelling can extract EBV‐DNA from ISF, and AuNWs can conduct electrical signals to flexible microfluidics for quantitative detection. This technology allows for minimally invasive monitoring of patients with EBV‐DNA‐related diseases and cancer metastases. In this complex MNs, hydrogel can improve the retention of nanomaterials in the skin, and nanomaterials can expand the sampling ability of hydrogels.

#### 4.1.3 Recent advances of MN patches for wound healing

Hydrogels with good biocompatibility are often used for wound healing. Recently, there was a study using separable MNs for promoting wound healing (Figure 4c). The MN tips containing black phosphorus (BP) nanoparticles and oxygen carrier are prepared by GelMA hydrogel, and the MN base is prepared by PVA.^112^ When the needle tips penetrate the tissue to absorb water, the contact area between hydrogel tips and PVA base will dissolve. Then, the needle tips containing BP and oxygen carrier will stay in the tissue, the PVA base can be removed. Under the irradiation of 808 NIR, the photothermal effect of BP results in the release of O_2_, to promote wound healing. BP nanoparticles can result in the release of O_2_, and the hydrogel needle tips can fix BP and release O_2_ around the wound. Compared with massive hydrogel scaffolds, nanomaterial composite hydrogel MNs can deliver drugs directly inside the wound. There is no need to experience the process of drug diffusion from the outside to the wound, which greatly improves the drug delivery efficiency. This is the first study to combine BP nanoparticles with hydrogel MNs for wound healing. It shows the great potential of nanomaterial composite hydrogel MNs in promoting wound healing.

### 4.2 Recent advances of injectable nanocomposite hydrogels

Injectable nanocomposite hydrogels can be prepared by adding nanomaterials to injectable hydrogels. Nanomaterials can adjust the structures and properties of hydrogels at the nano level and can also expand the functions of the system (such as PTT and PDT). Injectable hydrogels can effectively improve the retention effect of nanomaterials and make the composite systems have good plasticity to adapt to various biomedical application.^113^ One of the outstanding advantages of injectable nanocomposite hydrogels is that they can almost adapt and fill any application place (such as various of wounds). In injectable systems, nanomaterials generally blend with hydrogels through physical action. The interaction (mainly charge interaction, hydrogen bonding, and hydrophobic forces) between nanomaterials and hydrogels can improve several properties of systems, such as mechanical strength, stretchability, and adhesion. For example, an injectable nanocomposite hydrogel was developed by physically adding CNC NPs to a chitosan/β‐phosphate temperature‐sensitive gel.^35^ The injectable hydrogel was adapted to irregular wounds, and CNC NPs can improve the mechanical strength of the hydrogel through nano‐enhancement (hydrogen bonding and hydrophobic forces). Moreover, nanomaterials can even provide systems with many new properties such as antibacterial activity, electrical conductivity, and photothermal activity. For example, Huang et al. developed nanoparticles with photothermal activity, which can produce high temperature (50°C) and release NO gas under 808 nm laser. Subsequently, the nanoparticles were blended with methacrylate modified gelatin/hyaluronic acid grafting dopamine matrix, to obtain a photopolymerization injectable nanocomposite hydrogel. Under the 808 nm laser, the nanocomposite hydrogel has synergistic effect of PTT and gas therapy and has strong antibacterial activity.^40^ Moreover, mechanical properties are also important parameters and should be designed according to their uses. For example, the hydrogel used for bone repair has strong mechanical properties matching the bone (Young modulus ≈30–60 kPa),^32^ and the hydrogel used for skin repair has weak mechanical properties matching the skin (Young modulus ≈5–20 kPa).^40^ The mechanical strength of nanocomposite hydrogels can be set by adjusting the crosslinking degree of hydrogels or the ratio of nanomaterials to hydrogels.^35^ Nanomaterials can interact with hydrogels (hydrogen bonds, hydrophobic forces, and charge interactions) to enhance the mechanical properties. Injectable nanocomposite hydrogels can be widely used in biomedicine, such as cancer treatment, drug transport, and tissue regeneration.^79^, ^113^, ^114^

#### 4.2.1 Cancer treatment

Cancer has always been an extremely dangerous and deadly illness worldwide. Many emerging therapies based on nanotechnology have recently been developed, including PTT, photodynamic therapy (PDT), and gene therapy.^24^, ^114^ Tumor PTT based on nanomaterials has become a popular anti‐tumor method in recent years.^42^, ^47^ According to different light–mass interaction mechanisms in optical radiation, the photothermal conversion mechanism can be divided into two types stemming from nanomaterials, including nano metallic materials based on local plasmon heating and nano semiconductor materials with nonradiation relaxation.^115^ Both mechanisms can perform effective light‐to‐heat conversion. The photothermal mechanism of nano metal materials primarily involves the generation of thermal electrons under light radiation and the final photothermal conversion. Such nanomaterials include classic precious metal nanomaterials (such as Au NPs and Ag NPs) and emerging two‐dimensional plasmon materials (such as MXenes).^115^ The photothermal mechanism of nano‐semiconductor materials primarily involves electron diffusion under light radiation and composite carriers.^116^ The 2D BP is an example of these materials. The anti‐tumor effect of PTT is related to the photothermal conversion efficiency of nanomaterials.^117^ Compared to 0DM and 1DM, 2DM possesses outstanding advantages in light‐to‐heat conversion. First, under the same quality, 2DM exhibits a larger light absorption area that allows for more effective light‐induced heating. For certain 2DM, the light‐to‐heat conversion efficiency (PTCE) can even be as high as 100%. Second, 2DM exhibits a unique thickness‐dependent band gap. For example, by manufacturing BP nanosheets with different layers, the band gap of the BP can be adjusted to between 0.3 and 2 eV.^70^ Therefore, the 2DM's light radiation wavelength of the photothermal response can be expanded by combining multiple layers. Recently, many 2DM that possess good photothermal effects have emerged.^45^, ^50^, ^115^

**FIGURE 5:**
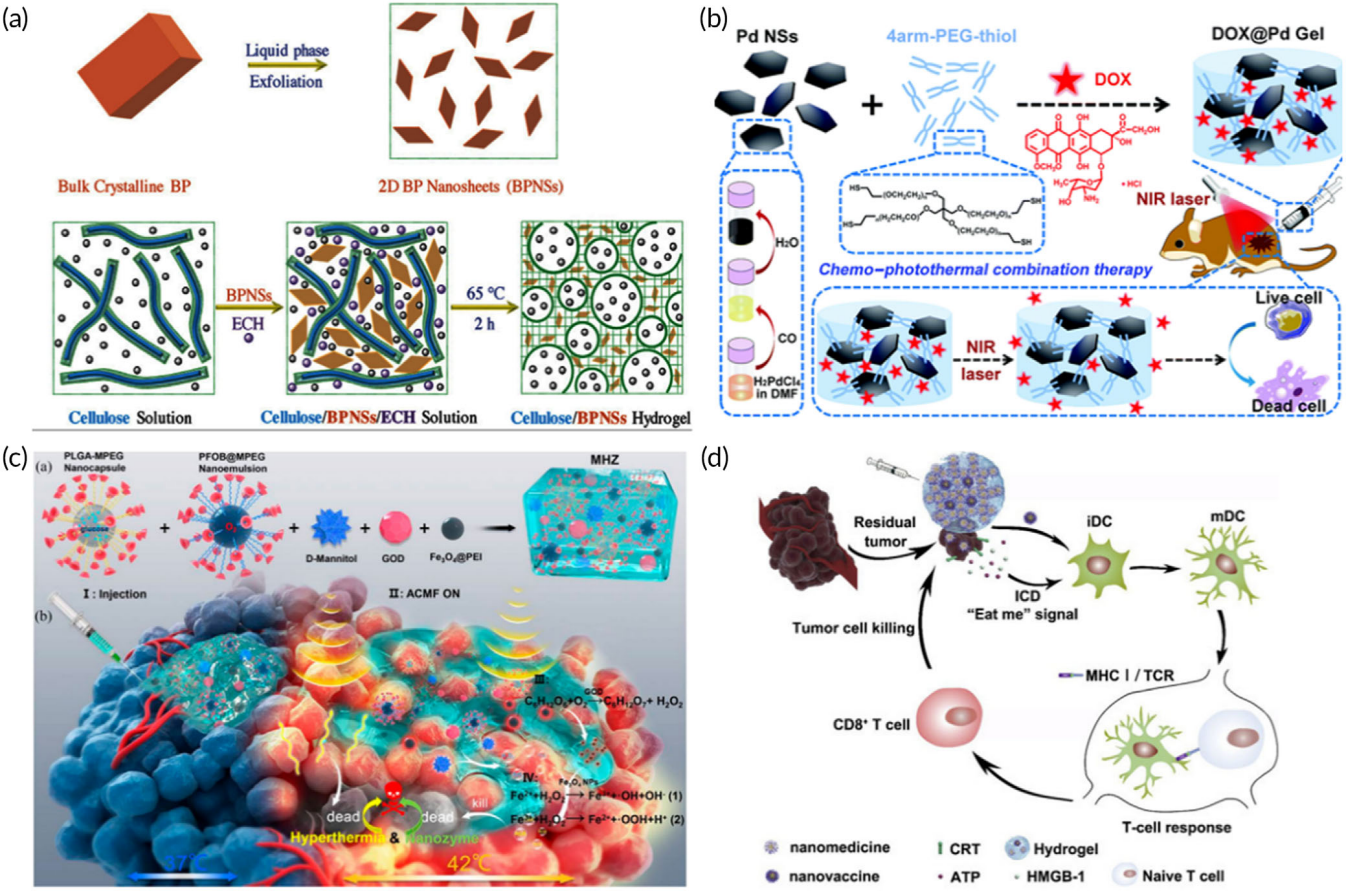
(a) Preparation of BP NSs composite hydrogels. *Source*: Adapted with permission from ref 45. Copyright 2018, WILEY‐VCH. (b) Diagrammatic sketch of DOX @ Pd hydrogel and the chemical‐photothermal combined treatment. The NIR cannot only activate 2D Pd NSs' PTT capability, but it can also promote the release of DOX within the gel. *Source*: Adapted with permission from ref 118. Copyright 2020, the Royal Society of Chemistry. (c) The working principle of MHZ gels. (a) Preparation path of MHZ gels. (b) Magnetocaloric response and the ROS mechanism for the synergistic treatment of tumor. *Source*: Adapted with permission from ref 119. Copyright 2019, American Chemical Society. (d) Diagrammatic sketch of tumor immunotherapy after hydrogel encapsulation of nano‐vaccine and nano‐drug. Continuous release of nano‐drug could achieve chemotherapy and promote the recruitment of host cells. At the same time, nano‐vaccines could deliver antigens to DCs and mature these cells. *Source*: Adapted with permission from ref 120. Copyright 2020, Elsevier

The research group of Professor Zhang Han of Shenzhen University developed an injectable nanocomposite hydrogel composed of cellulose and 2D black phosphorus nanosheets (BP NSs) (Figure 5a). This composite system can effectively treat tumors by PTT. In the composite system, 2D BP possesses extremely high photothermal efficiency to treat tumors, and the cellulose hydrogel can delay the degradation rate of BP.^45^ Free BP NSs easily settle in the physiological microenvironment, which inevitably leads to uneven photothermal effect. BP NSs were therefore dispersed in the hydrogel firstly in vitro. The nanocomposite hydrogel was then injected into the tumor via a syringe. Due to the large number of hydrogen bonds of cellulose, the hydrogel can still maintain a gel form after injection. Hydrogel and nanomaterials will not spread to the whole tumor. The hydrogel first produced short‐time local high temperature through photothermal action and then produced high‐temperature inhibition on the whole tumor through heat conduction. Although hyperthermia exerts a good therapeutic effect on tumors, it can be improved by combining hyperthermia with other treatment methods (chemotherapy, photodynamic therapy [PDT], catalytic therapy, and vascular rupture therapy) to construct a multi‐functional collaborative treatment platform.^50^, ^99^ Wang et al. reported an injectable thermosensitive hydrogel that incorporated nano‐sized titanium dioxide (B‐TiO2‐x).^37^ B‐TiO2‐x nanocrystals, which provide plentiful oxygen vacancies, enable the nanocomposite gel to exert both PDT and PTT abilities in response to NIR. Liang et al. designed an injectable nanocomposite hydrogel that combined PTT with vascular rupture for anti‐tumor therapy.^121^ Jiang et al. used palladium nanosheets (Pd NSs) as a cross‐linking agent, and they mixed this agent with four‐arm polyethylene glycol to obtain a novel nanocomposite injectable hydrogel (Figure 5b).^118^ The hydrogel encapsulates the antitumor medicine DOX to form DOX@Pd hydrogel. Pd NSs could respond to NIR, to combine chemotherapy with PTT for anti‐tumor therapy. Jin et al. loaded positively charged DOX and negatively charged PC10A onto the surface of MoS_2_ nanosheets to prepare PC10A/DOX/MoS_2_ nanoparticles. Then, the PC10A/DOX/MoS_2_ NPs were mixed into the gel to obtain an injectable nanocomposite gel system. The 2D MoS_2_ NSs within the hydrogel can be used as both photothermal agents and photodynamic agents to generate high temperatures and active oxygen under NIR. The prepared multifunctional PC10A/DOX/MoS_2_ composite injectable hydrogel can be used to treat tumors in combination with chemotherapy, PTT, and PDT therapy.^122^ In tumor thermal therapy, in addition to PTT, magnetothermal therapy based on nanomaterials also exhibits great application potential. Wu et al. designed a magnetic hydrogel nanozyme (MHZ) obtained by assembling PEGylated nanoparticles (Fe_3_O_4_@PEI NPs) in combination with cyclodextrin. The Fe_3_O_4_@PEI NPs were dispersed in the hydrogel to avoid precipitation in the tumor. The Fe_3_O_4_@PEI NPs in MHZ can produce a 42°C high temperature and hypertoxic active oxygen in response to magnetic field to achieve anti‐tumor (Figure 5c).^119^

Tumor immunotherapy based on injectable nanocomposite hydrogels also exhibits very good potential.^122^ Duong et al. developed an intelligent injectable nanocomposite gel loaded with nanocomposites possessing immunomodulatory factors.^123^ The hydrogel can be administered subcutaneously using minimally invasive techniques, and it can effectively locate and recruit immune cells. Liu et al. developed a thermally responsive hydrogel assembled with curcumin‐loaded polymer nanoparticles (Figure 5d).^120^ It can enhance the immunogenicity of the tumor by positioning and delivering nano drugs, and this gel enhances the immunity of anti‐tumor T cells by providing nano‐vaccines for the postoperative treatment of tumors. Additionally, immunotherapy can achieve synergistic treatment with other therapies. Jiang et al. designed multifunctional dendritic nanoparticles and injectable thermosensitive hydrogels, and they used them to build a local medicine transport platform that allowed for both chemotherapy and immunotherapy. Nanoparticles integrate the anticancer drug DOX and arginine‐rich molecules to achieve both chemotherapy and immunotherapy.^124^ While hydrogel can evenly disperse nanoparticles into the 3D structure to avoid the settlement of nanoparticles in the physiological microenvironment. Hydrogel can also act as an nanoparticles bank and store nanoparticles in the focal site. Moreover, hydrogel can slow release nanoparticles, effectively avoid the burst release, and improve the action time and effect of nanoparticles.

#### 4.2.2 Myocardial tissue engineering

Myocardial infarction (MI) is a dangerous disease that threatens the lives of individuals worldwide. Biomaterials used for myocardial repair must correctly mimic natural heart tissue. The designed materials must support the growth of myocytes, promote myocardial vascularization, and alter the myocardial microenvironment. The most important aspect is that they possess conductive properties that match endogenous bioelectric signals.^125^

**FIGURE 6:**
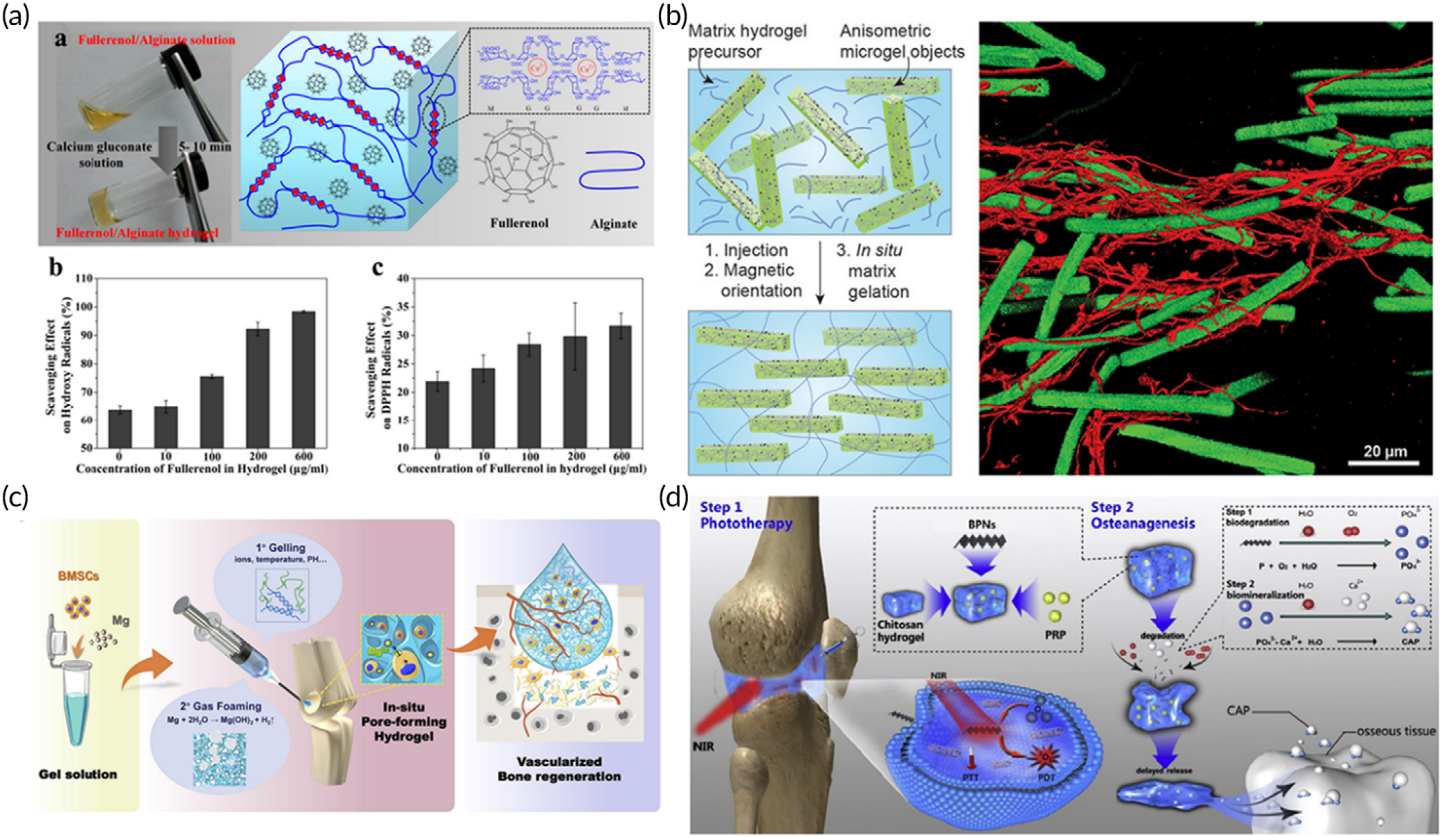
(A) Design and characterization of fullerenol/alginate gel. (a) Gelation and design of fullerenol/alginate gel. Clearance of fullerenol/alginate gel toward (b) hydroxy and (c) DPPH radicals. *Source*: Adapted with permission ref 53. Copyright 2017, American Chemical Society. (b) The working mechanism of Anisogel hydrogel. The cells strongly sense the resulting macroscopic unidirectional orientation, thus causing parallel nerves to stretch. *Source*: Adapted with permission from ref 131. Copyright 2017, American Chemical Society. (c) The BMSC and Mg particles were encapsulated in the hydrogel. *Source*: Adapted with permission from ref 132. Copyright 2020, Elsevier. (d) PRP‐chitosan thermal response hydrogel composite BP NSs can be used for the biological treatment and phototherapy (PTT, PDT) of rheumatoid arthritis. *Source*: Reproduced with permission from ref 133. Copyright 2020, Elsevier

The conductivity of materials exerts an important effect on the recovery of myocardial function. Researchers have combined injectable reverse thermal gels (RTGs) with CNTs or Au NPs to develop a biomimetic conductive composite gel system that can be used for myocardial repair.^126^, ^127^ Bao et al. synthesized a conductive injectable hydrogel, and they then incorporated GO into the hydrogel to provide the hydrogel with excellent conductive properties. This hydrogel loaded with adipose‐derived stem cells (ADSCs) was used to improve cardiac function in the MI area.^128^ Additionally, myocardial infarction can cause myocardial ischemia and based on this, pro‐vascularization is necessary during treatment.^129^ Wang et al. added a nanocomposite containing a plasmid encoding eNOS to the material system developed by Bao et al. The composite can improve the expression of eNOS, promote the myocardial angiogenesis, and improve the myocardial function.^130^ In addition to conducting electricity and promoting vascularization, the ROS environment at the site of myocardial infarction can also limit the therapeutic effect of myocardial infarction, particularly during stem cell implantation treatments. Therefore, it is necessary to develop materials that can resist ROS. Hao et al. compounded fullerenol nanoparticles with alginate hydrogels to design a cell transport platform with antioxidant activity (Figure 6a). ROS can easily adhere to the electron‐deficient position on the surface of fullerenol nanoparticles, further the ROS on the adjacent electron‐deficient position may induce the destruction of ROS via transferring electron to the fullerenol cage. This material system could effectively reduce ROS generated during myocardial infarction, thus effectively increasing the retention and survival rate of the stem cells to enhance angiogenesis at the site of myocardial infarction.^53^

#### 4.2.3 Nerve tissue engineering

Both nerve tissue and myocardium are electroactive tissues. Additionally, nerve tissue possesses a high orientation. Therefore, the repair of nerve tissue should not only consider the mechanical property and the bio‐electrochemical signals, but more importantly, it should allow the nerve tissue to be capable of directional growth and healing. Anisogel gel is an injectable nanocomposite hydrogel that is minimally invasive and possesses the ability to guide the unidirectional growth of cells and nerves. Rose et al. incorporated ultrasmall superparamagnetic iron‐oxide nanoparticles (USPIOs) into microgels and then dispersed the microgels into injectable gel precursors to develop a novel highly oriented injectable mixed gel (Anisogel gel, Figure 6b).^131^ After minimally invasive injection of the material into the injury site, the microgels can be controlled to be aligned in a specific orientation through an extracorporeal magnetic field. Then the gel precursor can be cross‐linked to fix the microgels inside the matrix hydrogel. Even if the external magnetic field is removed, the microgels can still maintain a specific orientation to allow the cells to strongly sense the resulting macroscopic unidirectional orientation characteristics. Finally, the neurons are induced to grow in one direction in parallel to repair the damaged nerve tissue. Compared with other hydrogels before implantation,^134^ injectable nanocomposite hydrogel in situ has many advantages, such as can be minimally invasive, can adapt to various irregular shape wounds, can load cells for injecting, and can regulate the cell growth direction through external magnetic field. Subsequently, Omidinia et al. used magnetically responsive short fibers containing USPIOs to prepare another Anisogel gel. Compared to the added microgels of Rose et al., the magnetically responsive short fibers were prepared using a simple and effective high‐throughput method. The size of the short fibers could be easily manipulated and defined.^135^ Fibroblasts and nerve cells can achieve unidirectional growth and extension in Anisogels gel. Anisogel gel is the first biomaterial that can achieve an extremely controlled as well as ordered structure in situ to allow for the directional growth of cells and nerves after minimally invasive injection. It is of pioneering importance and possesses great application potential for nerve repair, particularly in the field of spinal cord repair. These studies were currently still in its in vitro research phase and can induce the directional growth of nerve cells in vitro. However, in vivo studies have yet to be further verified. As static magnetic fields cannot penetrate very deep into human tissue, which is an important reason for limiting their application in vivo.

#### 4.2.4 Bone tissue engineering

Injectable nanocomposite hydrogels can fill irregular bone defects with minimally invasive surgery and promote bone tissue regeneration. The porous structure of the hydrogel is conducive to cell proliferation and nutrient delivery.^136^ Hydrogels for bone repair require strong mechanical properties, while nanomaterials can enhance the mechanical properties of hydrogels through nano enhancement (hydrogen bonds, hydrophobic forces or charge interactions). Nanomaterials such as bioactive glass, calcium phosphate, and Mg nanoparticles, can promote the vascularization of bone tissue and osteogenic differentiation. The current treatment strategy possesses two main approaches: (1) injection of the nanohydrogel loaded with stem cells and (2) injection of nanocomposite hydrogels without cells and growth factors. The injection of nanocomposite hydrogels loaded with stem cells is mainly considered to induce in situ osteogenic differentiation of stem cells. Tang et al. developed an injectable hydrogel containing Mg particles (Figure 6c).^132^ H_2_ formed by the degradation of Mg will build a macroporous structure in the hydrogel to enhance the diffusion of oxygen and nutrients, thus increasing the survival rate of cells. Additionally, Mg^2+^ can promote in situ osteogenesis differentiation of BMSCs encapsulated in the gel.^132^ As for the second point, the key to injecting nanocomposite hydrogels without stem cells and growth factors is to recruit natural cells to achieve bone regeneration. Cui et al. designed an injectable chitosan gel containing 2D nanoclay. 2D nanoclay (overall negative charge) has charge interaction with the gel matrix (positive charge). Therefore, the 2D nanoclay can greatly increase the Young's modulus of the gel from 10 kPa to over 60 kPa, which is more suitable for bone repair. The nanoclay may lead to interconnected micropore structures of hydrogel, which can recruit natural cells and promote bone repair without delivering other therapeutic agents or stem cells.^32^ Wu et al. incorporated copper‐containing bioactive glass nanoparticles into chitosan composite silk fibroin hydrogel to develop a cell‐free injectable platform for bone repair.^137^ The hydrogel can effectively enhance bone vascularization and the deposition of mineralized collagen.

#### 4.2.5 Cartilage tissue engineering

As articular cartilage lacks blood vessels, nerves, and lymphatic networks, its self‐repairing ability is limited. Injectable hydrogels can effectively reduce the risk of infection and secondary trauma through minimally invasive injection. Hydrogels for cartilage repair also require strong mechanical properties. Incorporating nanomaterials into injectable hydrogels can effectively improve the mechanical strength of the gels and can facilitate the sustained delivery of chemical drugs, ultimately improving practicality for cartilage repair.^138^ Wang et al. incorporated rifampin MSNs and dendritic polymer template silver nanoparticles (G3‐Ag) into hydrogels to develop injectable biomimetic gels for cartilage repair and anti‐infection. MSNs and G3‐Ag can improve the mechanical properties of hydrogels. Additionally, rifampicin encapsulated in MSNs can achieve long‐term release.^139^ Compared to antibiotic therapy, photodynamic therapy (PDT) based on nanomaterials is also a hot topic that has recently been explored. In the process of cartilage formation and differentiation, ROS produced by PDT are necessary for cell survival, proteoglycan secretion, and cartilage formation. Several studies have cross‐linked 0DM quantum dots (carbon dot nanoparticles [CD NPs] and cadmium selenide quantum dots [CdSe QDs]) to collagen using the cross‐linking agent genipin, and developed injectable nanocomposite hydrogels for cartilage repair.^18^ CD NPs or CdSe QDs not only significantly improve the mechanical properties of collagen hydrogels (through hydrogen bonds, hydrophobic forces or charge interactions) but can also generate ROS through PDT to promote the directional differentiation of BMSCs into cartilage, ultimately accelerating the regeneration of cartilage. Recently, a synergism has been observed between PDT and PTT regarding joint disease treatment. Pan et al. incorporated black phosphorus nanosheets (BP NSs) into thermally responsive injectable chitosan hydrogels to develop a therapeutic platform for the treatment of cartilage defects caused by rheumatoid arthritis (Figure 6d). The 2D BP produces local heat (PTT) and ROS (PDT) under NIR, which cannot only remove hyperplastic synovial tissue but also stimulate the regeneration of cartilage injury. Concurrently, the degradation products of BP can provide sufficient raw materials for osteogenic differentiation.^133^

#### 4.2.6 Skin tissue engineering

**FIGURE 7:**
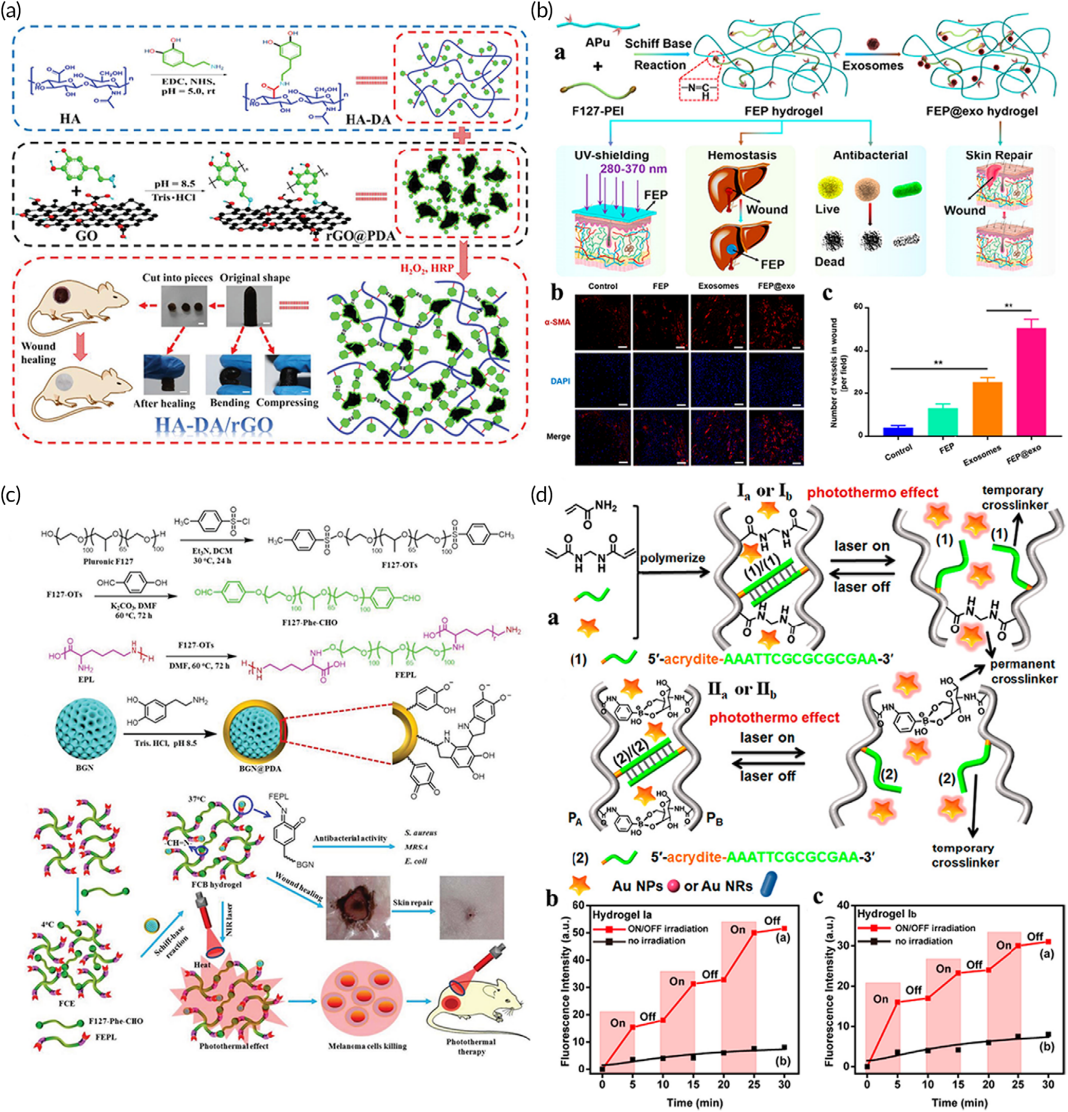
(a) The design route and the original, curved, compressed, and self‐healing forms of rGO nanocomposite gel and their application in full‐thickness skin wound healing. Scale bar: 5 mm. *Source*: Adapted with permission from ref 69. Copyright 2019, WILEY‐VCH. (b) The preparation process of injectable thermosensitive hydrogel (FEP@exosomes) and its antibacterial ability, rapid hemostasis, anti‐ultraviolet activity, and diabetes wound repair properties (a). FEP@exo hydrogel can stimulate angiogenesis in diabetic wound tissue. Immunofluorescence staining of α‐SMA (b) and of neovascularization (c) on Day 7. Scale bar: 20 μm. *Source*: Adapted with permission from ref 141. Copyright 2019, American Chemical Society. (c) The synthesis route of FCB hydrogel and its effect in cancer treatment and wound repair. *Source*: Adapted with permission from ref 142. Copyright 2019, WILEY‐VCH. (d) (a) Gold NPs or NRs are introduced to create two DNA‐based hydrogels. The hydrogels can be used to develop light‐controlled mechanical properties and to promote shape memory, light‐controlled drug release, and self‐healing through on/off irradiation. (b, c) Controlled release of DOX from hydrogels with (Curve a) or without laser irradiation (Curve b). *Source*: Adapted with permission ref 143. Copyright 2019, American Chemical Society

Skin damage is often accompanied by bacterial infections that cause secondary wound deterioration.^40^ The shape and depth of the skin wounds are often irregular, and it is difficult to fully fit the wound even after cutting the gels. For injectable nanocomposite hydrogels, inject the gel‐precursor to the wound surface first, and then formed hydrogel by temperature, pH, light, and so on. The hydrogels can completely match various irregular wounds to achieve good treatment effect. Gao et al. mixed polydopamine nanoparticles (PDA NPs) with modified chitosan to prepare an injectable gel for combating bacterial infections and promoting wound healing.^140^ PDA NPs contain the potent antibiotic ciprofloxacin (Cip). Near‐infrared light irradiation can promote the release of Cip, and at the same time, it can activate PDA NPs to produce a photothermal effect (PTT). The local heat can lead to the destruction of bacterial integrity. The hydrogel system thus achieved antimicrobial activity through photothermal effect and antibiotics. Ordinary skin injuries can heal smoothly within 1 or 2 weeks. However, full‐thickness skin injuries are often difficult to repair and can even endanger human lives. Moreover, studies have shown that the tissue adhesion, electrical conductivity, antioxidant activity, and hemostatic ability of skin dressings are also essential for wound healing. Liang et al. designed an injectable hydrogel made from HA‐grafted‐dopamine and rGO NSs that can be used to enhance the regeneration of full‐thickness skin damage (Figure 7a).^69^ Dopamine provides the hydrogel with antioxidant activity, tissue adhesion ability, hemostatic functionality, and photothermal antibacterial properties. rGO provides the hydrogel with good electrical conductivity.

Compared to ordinary skin wounds, the healing of diabetic wounds is more challenging. Diabetes can cause microvascular dysfunction that leads to ischemia and hinders wound healing. Therefore, for diabetic wounds, enhancing angiogenesis and restoring revascularization are important to accelerate wound healing. Exosomes are a bioactive nanovesicles secreted by stem cells and are negatively charged on surface. Exosomes usually possess a diameter of 50–200 nm. Compared with stem cells, they elicit a lower immune response and possess higher stability and storability.^144^ Studies have shown that exosomes exhibit an extremely high potential for promoting wound healing. Min Wang et al. designed an injectable temperature‐sensitive gel dressing to promote diabetic wound healing and angiogenesis (Figure 7b). The hydrogel system exhibits effective antibacterial activity, self‐healing, tissue adhesion, rapid hemostatic ability, and good ultraviolet shielding performance, and the ability to continuously release exosomes in response to the pH of diabetic wounds.^141^ Exosomes bind to the PEI in the hydrogel by electrostatic action. Under the condition of slightly acidic pH, the dissociation of binding sites will be caused by free charge (H^+^), which leads to the release of exosomes from hydrogel. The released exosomes accelerate wound repair primarily by enhancing the angiogenic ability of diabetic wounds. Exosomes contain stem cell‐specific proteins and nucleic acids and are capable to partially mimic stem cell function. They can recruit vascular endothelial cells and promote their proliferation, migration, and formation of neovascularization, improve blood flow perfusion and oxygen supply, thus promoting wound healing.

Surgery to treat skin tumors can also cause skin damage. Compared to ordinary skin injuries and diabetic wounds, the additional consideration of skin tumor surgical trauma is to eliminate possible residual tumor cells to prevent recurrence. Therefore, injectable hydrogels applied to skin tumor surgical wounds should not only promote wound healing and possess antibacterial activities, but they also should help to prevent residual tumor recurrence. Due to its high efficiency, low toxicity, and noninvasive nature, PTT is commonly used in the treatment of skin tumors. Zhou et al. developed a multifunctional bioactive nanocomposite injectable hydrogel that not only promotes wound healing and resistance to multidrug resistant bacteria but also effectively eliminates skin tumors (Figure 7c). The polydopamine‐functionalized bioactive glass nanoparticles (BGN@PDA) incorporated into the antibacterial hydrogel exhibited excellent photothermal properties (PTT) under near‐infrared laser irradiation. The local heat generated by PTT can effectively kill bacteria as well as tumor cells (>90%).^142^ In addition to PTT, PDT is also regarded as a noninvasive treatment for skin tumors that can effectively avoid secondary trauma. Wang et al. integrated black titanium dioxide (B‐TiO_2_) nanoparticles into a chitosan matrix to develop an injectable temperature‐sensitive nanocomposite hydrogel. The gel cannot only promote the regeneration of skin tissue but can also mimic the effects of PTT and PDT to effectively inhibit skin tumors (Table 2).^37^

**TABLE 2:** Representative nanocomposite hydrogels for biomedical applications

Composite systems	Application	Nanomaterials	Hydrogels	Properties	Mechanism	References
Nanocomposite hydrogel micro‐needle patches	Intelligent insulin delivery	Polymer nanovesicle NPs	Cross‐linked hyaluronic acid (HA‐MA)	Painless and intelligent regulation of blood glucose balance	Nanovesicle can amplify the blood glucose signal	33
		Polymer nanovesicles NPs	HA‐MA	Response to H_2_O_2_ and painless drug delivery	The nanoparticles can response to H_2_O_2_	99
		Polymer nanovesicles NPs	HA‐MA	Response to hypoxic and H_2_O_2_, reduce inflammation	The nano‐particles can response to hypoxic and H_2_O_2_	109
		Nano‐Micelles NPs	PVA	Response to H_2_O_2_ and painless drug delivery	The nano‐particle can eliminate H_2_O_2_, thus reducing inflammation	110
	Sampling interstitial fluid	Nano‐Capsules NPs	Alginate	Painless Sample interstitial fluid and immunocyte	Molecular adjuvants and specific antigens were encapsulated in nanocapsules, can capture specific immune cells	97
		Au NWs	Polymethyl vinyl ether‐salt‐maleic acid	Real‐time quantitative assay of EBV‐Cf DNA	Hydrogel swells, Au NWs can conduct electricity	2
	Wound healing	BP NPs	GelMA	Promote wound healing	The photothermal effect of BP results in the release of O_2_, to promote wound healing.	112
Injectable nanocomposite hydrogels	Cancer treatment	BP NSs	Cellulose	PTT	Local high temperature killed the tumor cells	45
		B‐TiO2‐x NPs	Chitosan	PDT and PTT synergistic therapy	Reactive oxygen species and high‐temperature anti‐tumor	37
		Fe_3_O_4_@PEI NPs	Poly(lactic‐*co*‐glycolic acid) and perfluorooctyl bromide	Magnetic heat and ROS synergistic therapy	Fe_3_O_4_@PEI NPs can produce high temperature and ROS in response to magnetic field	119
		Dendritic NPs	Poly(d,l‐lactide‐*co*‐glycolide)‐poly (ethylene glycol)	Chemotherapy and immunotherapy synergistic therapy	Dendritic NPs integrate the anti‐tumor drug DOX and arginine‐rich molecules	124
	Myocardial tissue engineering	Fullerenol NPs	Alginate	Increase stem cell survival, enhance angiogenesis	Fullerenol NPs can eliminate ROS, thus increasing stem cell survival	53
	Nerve tissue engineering	USPIO NPs	Star‐PEG‐A	Minimally invasive injection, can induce directional cell growth	In situ gelation, USPIO NPs can response to extracorporeal magnetic field	131
	Bone tissue engineering	Nanoclay NSs	Methacrylated glycol chitosan	High mechanical strength, without loading cells and growth factors	Nanoclay NSs have charge interaction with the gel matrix, the complex can recruit native cells for bone regeneration	32
	Cartilage tissue engineering	BP NSs	Chitosan	Injectable, PTT/PDT multifunctional treatment platform	Chitosan has temperature‐sensitive property, PTT can remove hyperplastic synovial tissue, PDT can stimulate the regeneration of cartilage injury	133
	Skin tissue engineering	Exosomes NPs	Aldehydepullulan (APu) and Pluronic F‐127	Injectable, in response to the pH releasing exosomes	F‐127 has temperature‐sensitive property, the electrostatic action of exosomes‐PEI is destroyed in acidic environment	^141^
Self‐healing nanocomposite hydrogels	Controlled drugs release	Au NPs or Au NRs	Polyacrylamide	Laser‐controlled drugs release	Photothermal effect resulting in the crosslinking decrease of hydrogel to release the drug	149
	Bionic electronic skin	Proanthocyanidin (PC)/rGO NPs	Glycerol‐PVA‐borax	Super‐stretchability, instant self‐healing ability, simulating the touch of natural skin	Glycerol chelates with borax and forms hydrogen bond with PVA. PC/rGO provides electrical conductivity for composites	151
	Glucose sensor	CeO_2_/MnO NPs	Quaternized chitosan composite oxidized dextran	Rapid and sensitive response to glucose, self‐healing property	CeO_2_/MnO_2_ NPs act as electrocatalytic medium, Schiff base bond between quaternized chitosan and oxidized dextran	57
	Strain sensor	GO NSs and Ag NWs	PVA/Ca^2+^	Strong mechanical toughness, self‐healing property, conductivity	Ag NWs endow the sensor system with high conductivity, The PVA‐Ca^2+^ ‐GO network forms a large number of hydrogen bonds	156
	Bioactuator	Boron nitride NSs	Poly(acrylamide‐*co*‐maleic anhydride)	Self‐healing property, tensile strength and toughness, frost and high‐temperature resistance	A large number of irregular hydrogen bonds formed by glycerol‐polymer‐water network	55
Bioimaging nanocomposite hydrogels	Antitumor bioimaging	Ag_2_S NPs	Coiled‐coil polypeptides	Monitor hydrogel degradation at tumor sites dynamically	Ag_2_S NPs can perform photoacoustic and fluorescence bioimaging	162
	Tissue engineering bioimaging	USPIOs NPs	Ellulose nanocrystal/dextran	Noninvasive monitoring of bone	USPIOs can response to in vitro MRI	97
	Cell behavior bioimaging	Ag NPs	Polymeric DNAs with cytosine‐rich sequences	Monitoring active oxygen species and nitrogen substances in living cells	Strong oxidant decreased the red fluorescence and enhanced the green fluorescence of Ag NPs	168

*Note*: NPs (nanoparticles), NRs (nanorods) belong to 0DM; NWs (nanowires) belong to 1DM; NSs (nanosheets) belong to 2DM.

### 4.3 Recent advances of self‐healing nanocomposite hydrogels

Self‐healing hydrogel means that the material can repair properties to their original state after being damaged, which could prolong the lifespan of the material and reduce its unreliability.^106^ Referring to the self‐healing mechanism, the self‐healing behavior is mainly induced by the reversible interaction of the hydrogel itself (dynamic chemical bonds and noncovalent interactions).^101^, ^145^ There are many dynamic chemical bonds, such as C—N bond (acyl hydrazone bond and imide bond), B—O bond (phenyl borate), C—C/C—S bond (reversible radical reaction), Schiff base bond and disulfide bond. There are also many dynamic noncovalent interactions, such as multiple hydrogen bond interaction, π–π stacking, ion interaction (metal coordination), host–guest interaction, and hydrophobic interaction.^58^, ^101^, ^106^, ^145^ In recent years, self‐healing hydrogels have exhibited great potential to become brittle hydrogel substitutes based on their durability and long‐term stability.^106^ However, depending on their intended use, self‐healing hydrogels also must include a variety of properties such as electrical conductivity, photosensitivity, adhesion, and appropriate mechanical strength. Nanomaterials are small and possess various characteristics such as conductivity, light sensitivity, and pH response.^16^, ^146^ Therefore, a promising method is to incorporate nanomaterials into self‐healing hydrogels to develop nanocomposite hydrogels possessing multiple functions and self‐healing ability. Due to the interaction of polymer‐nanomaterials (such as charge interaction, hydrogen bond and hydrophobic interaction), nanomaterials can also enhance the self‐healing ability of hydrogels.^106^ Self‐healing nanocomposite hydrogels exhibit huge application potential in controlled drugs release, electronic skin, and biosensing.^147^

#### 4.3.1 Controlled drugs release

Self‐healing nanocomposite hydrogels can control the release of medicines through dynamic networks on/off.^148^ Wang et al. designed DNA‐based shape‐memory self‐repairing hydrogels loaded with gold nanoparticles (Au NPs) or nanorods (Au NRs).^149^ Au NPs or AuNRs make hydrogels have sensitive laser‐controlled drugs release capabilities as well as mechanical strength adjusted by light (Figure 7D). Nanomaterials endow the hydrogels with self‐healing and drug release performance of photo‐response. The Au NPs (λ = 532 nm) or Au NRs (λ = 808 nm) heated the hydrogel by photothermal effect, resulting in dehybridization of the DNA duplexes. The crosslinking of the hydrogel decreased to release the drug. Chen et al. prepared a temperature‐sensitive nanogel and then formed a nanocomposite hydrogel with a hydrophilic copolymer that can rapidly gel at physiological pH.^150^ The hydrogel not only has self‐healing ability, but also shows multiresponsiveness to pH, glucose, H_2_O_2_, and temperature. The hydrogel system has huge potential for controlled drugs release.

#### 4.3.2 Bionic electronic skin

**FIGURE 8:**
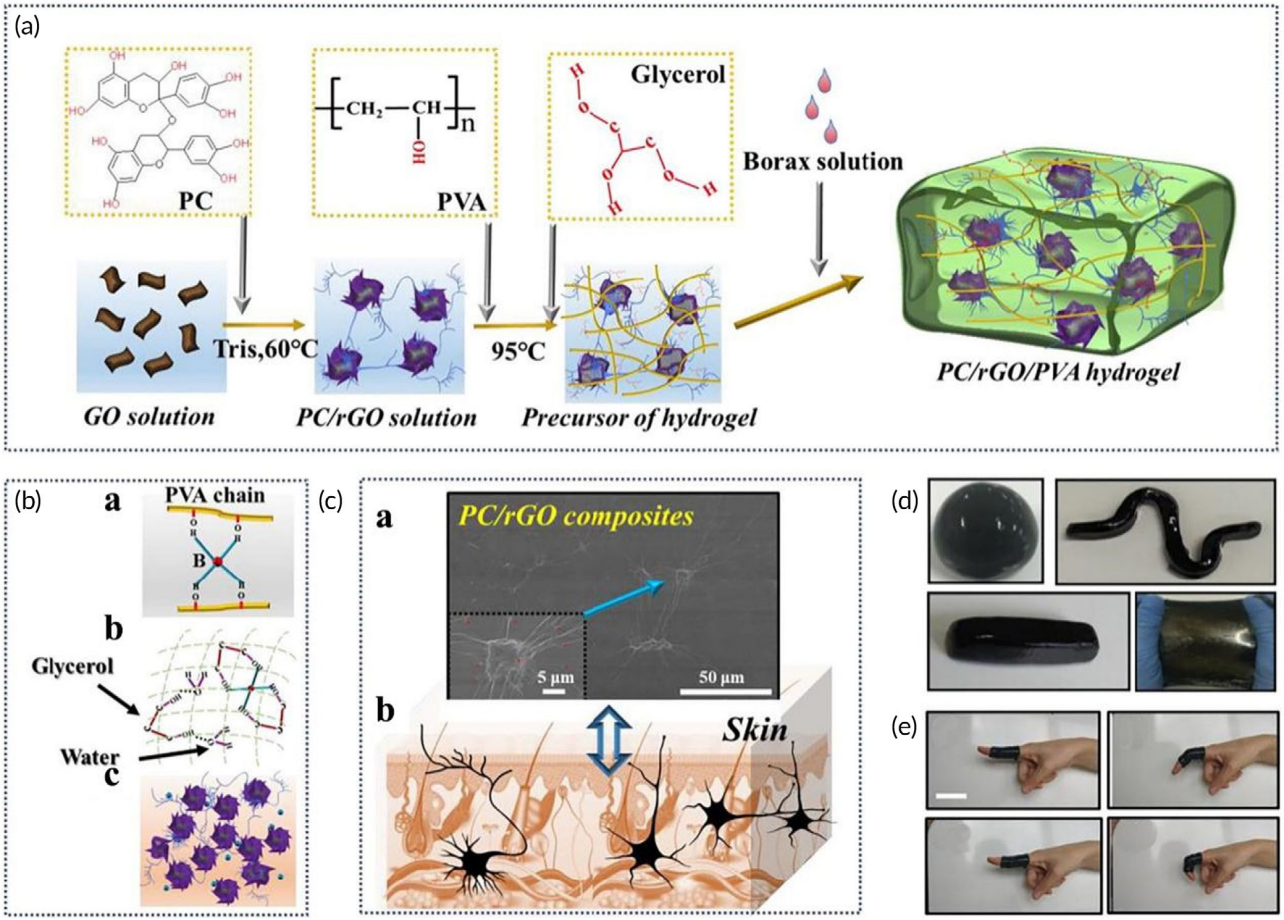
(a) Design diagrammatic sketch of a PC/rGO/PVA gel. (b) (a) The PVA meshwork, (b) the borax‐glycerol‐water meshwork, and (c) the neuroid PC/rGO meshwork. (c) (a) The SEM image of the PC/rGO meshwork, (b) the distribution of tactile nerves in the skin. (d) The PC/rGO/PVA gel can be modeled into multiple shapes. (e) The gel can dynamically adapt to finger movements (scale bar: 5 cm). *Source*: Reproduced with permission from ref 151. Copyright 2020, Elsevier

The materials used to prepare the electronic skin must possess good toughness, self‐healing properties, and electrical conductivity to allow them to withstand wear and convert external pressure and strain stimuli into electrical signals for analysis and detection. Pan et al. incorporated proanthocyanidin (PC)/rGO composites and neuro‐like nano‐networks into the glycerol‐PVA‐borax gel and developed an electronic skin composed of Bionic tactile hydrogel (PC/rGO/PVA).^151^ Glycerol chelates with borax and forms hydrogen bond with PVA and forms a borax‐glycerol‐water network, giving hydrogel super stretchability, super adaptability and instant self‐healing ability. (Figure 8). Additionally, the PC/rGO composite nano‐network allows the hydrogel to perfectly simulate the touch of natural skin and can precisely perceive skin changes, expressions of the face, and phonation. PC/rGO nanoparticles can provide electrical conductivity for composite materials and can transmit and recognize external signals through the structure and electrical conductivity of hydrogels, simulating the touch of natural skin. Lin et al. prepared an Ag/TA@CNC nano‐hybrid, and they then mixed it with PVA gel to prepare a bionic tactile hydrogel.^152^ This hydrogel integrates a variety of excellent properties, including rapid self‐healing ability, super stretchability, enhanced electrical conductivity, super compliance, self‐adhesiveness, and good antibacterial ability, mainly with the action of dynamic borate bond and hydrogen bond. Moreover, when the hydrogel is assembled inside the sandwich structure, it can sense various motion haptics such as large joint bending and subtle facial expressions, pronunciation, and breathing. This bionic material is sufficient to simulate various tactile functions of the human skin.

#### 4.3.3 Glucose sensor

**FIGURE 9:**
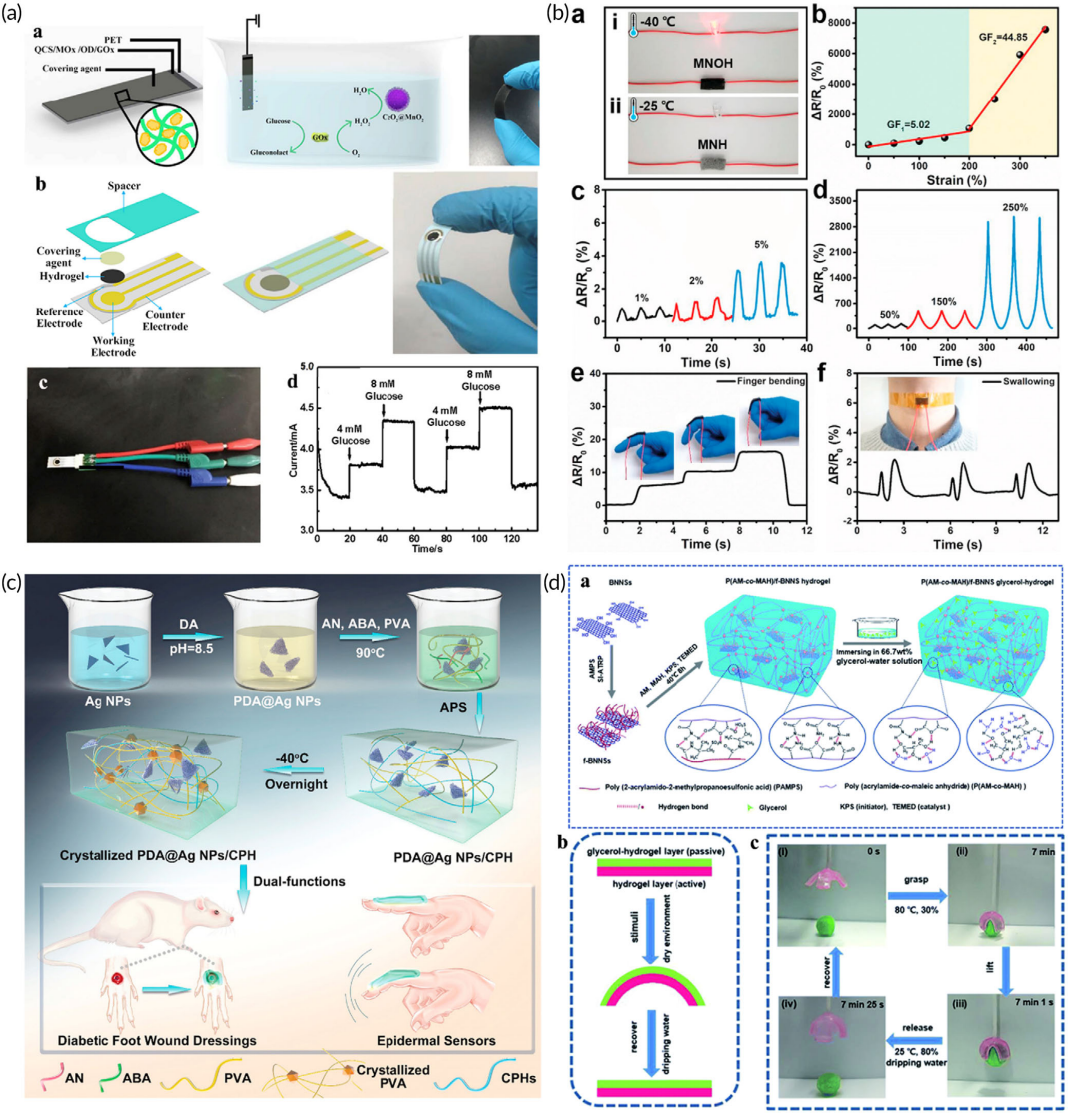
(A) (a) Diagrammatic sketch and photograph of a glucose electrode. (b) Schematic diagram of the construction of glucose sensors. (c) Physical photographs of the assembled glucose sensor. (d) The sensor can respond to the repetitive addition of 4 and 8 mM glucose. *Source*: Adapted with permission from ref 57. Copyright 2020, Elsevier. (b) (a) Gels in which partial water is replaced (MNOH) are more resistant to low temperatures than pure hydrogels (MNH). (b) MNOH‐based sensors' strain–resistance correlation curves. Sensors resistance responds to small strains (c) and to large strains (d), respectively. The sensors could still respond to fingers bending (e) and to throat movement (f) after being stored at −40°C for 6 h. *Source*: Adapted with permission from ref 49. Copyright 2019, WILEY‐VCH. (c) The synthesis procedure of PDA@Ag NPs/CPH hydrogels and their application in monitoring motions and diabetic wound repair. *Source*: Adapted with permission from ref 153. Copyright 2019, WILEY‐VCH. (d) (a) The design routes for P(AM‐*co*‐MAH)/f‐BNNS glycerol‐hydrogels. (b) Response braking process of the double‐layer gel. (c) Grab, lift, and release triggered by different temperatures. *Source*: Adapted with permission from ref 55. Copyright 2019, the Royal Society of Chemistry

Blood glucose monitoring is very necessary for diabetic patients. A continuous glucose monitoring system (CGMS) can monitor the blood sugar level of patients with severe diabetes to facilitate the accurate delivery of insulin to patients. The glucose sensor is one of the key components of the CGMS. Current commercial glucose sensors do possess certain defects such as being prone to mechanical damage, lack of durability, and leakage of contents. Liang et al. designed a self‐repair hydrogel made of quaternized chitosan composite oxidized dextran (Figure 2f). Then, CeO_2_/MnO_2_ nanoparticles loaded with GOx were connected to the gel through covalent action to serve as an electrocatalysis medium (Figure 9a).^57^ An additional covering agent was concurrently applied to the hydrogel to av CeO_2_/MnO_2_ NPs run‐off. The gel was then covalently bonded to a flexible chip to form a flexible glucose sensor. Since the CeO_2_/MnO_2_ NPs act as electrocatalytic medium, the sensor exhibits a rapid and sensitive response to glucose (*t* < 3 s). Due to the reversible Schiff base bond between quaternized chitosan and oxidized dextran, the hydrogel exhibits strong self‐repair properties that allow the sensor to adapt to various deformations and damage. The hydrophilic polymer network and self‐healing function greatly improve the sensitivity and service life of the glucose sensor, and the sensor can even work continuously for more than 30 days in vitro. In addition, study has been successfully examining glucose in oral saliva by nanocomposite hydrogel combined with GOx.^154^ These studies show that nanocomposite hydrogels combined with GOx has great potential in noninvasive detection of glucose.

#### 4.3.4 Strain sensor

Any hydrogel used as a skin strain sensor must possess good toughness, conductivity, and self‐repair ability to allow for long service life and good electrical signal transmission ability under external stress.^155^ Yang Liu et al. designed a nanocomposite hydrogel with a layered structure of “brick and mortar” that possessed rich dynamic interactions, and they used it as a strain sensor.^156^ In the hydrogel, “brick” materials were formed by GO NSs and Ag NWs, and “mortar” materials were formed by PVA/Ca^2+^. Ag NWs endow the sensor system with high conductivity and GO NSs provide the system with rich functional groups. PVA endows the entire sensor system with extremely strong mechanical toughness and self‐healing properties through many hydrogen bonds and polymer chain movements. Moreover, Ca^2+^ can form a powerful and reversible coordination bond with PVA and with GO, thus greatly improving the self‐repair capability of the gel. Moreover, when the ambient temperature is lower than zero, hydrogels that use deionized water as a dispersant are likely to freeze and thus experience performance degradation. Even at room temperature, such hydrogels will lose water due to evaporation, thus resulting in a poor life cycle. Therefore, Liao et al. developed a 2D MXene nanocomposite organic hydrogel (MNOH) with antifreeze, self‐healing, and conductive properties by using ethylene glycol to supersede the partial water of the gel.^49^ MXene is a 2D material composed of transition metal carbides or carbonitrides that can be used to prepare nanocomposite hydrogels with enhanced conductivity and mechanical properties. The hydrogel has excellent antifreeze properties (−40 °C), can retain moisture for a long period of time (8 days), possesses outstanding self‐repair capability, and exhibits mechanical capability. Additionally, the hydrogel can be crafted into wearable strain sensors possessing a relatively wide strain range at extremely low temperatures (Figure 9b).

Strain sensors can be prepared to monitor various human activities, such as finger movement, pulse frequency, and various types of breathing.^157^, ^158^ Additionally, to expand the functions and uses of the sensor, Zhao et al. developed a multifunctional nanocomposite hydrogel by assembling polydopamine‐modified Ag NPs with polymers (Figure 9c). The hydrogel possesses adjustable mechanical as well as electrochemical abilities, excellent self‐repair capability, and repeatable adhesion. It can be used to prepare strain sensors to monitor various motions in real‐time.^153^ Moreover, the hydrogel system can also promote angiogenesis, accelerate collagen deposition, anti‐infection, so as to repair diabetic wounds. The developed nanocomposite hydrogel combines the functions of tissue regeneration and biosensing, and it can likely achieve dynamic monitoring of human movement while repairing diabetic wounds.

#### 4.3.5 Bioactuator

Self‐healing nanocomposite hydrogels exhibit good application potential in bioactuators.^159^ Their flexibility and self‐healing properties provide actuators with enhanced performance and longer service life. Guo et al. introduced 2D boron nitride NSs into synthetic polymer hydrogels and then performed glycerin‐water treatment to successfully prepare a self‐healing glycerin‐hydrogel (Figure 9d).^55^ A large number of irregular hydrogen bonds formed by glycerol‐polymer‐water network not only make the gel exhibit excellent self‐healing property, tensile strength and toughness, but also destroy the formation of ice crystals at low temperatures, improve the freezing resistance of hydrogel (−45°C), and prevent the water from evaporating at high temperatures (60°C). Even if stored at −45°C for 3 months, the hydrogel still possesses excellent self‐healing property. The designed double‐layer hybrid hydrogel based on glycerin‐hydrogel and pure hydrogel can be used as a humidity‐temperature dual‐response actuator. Qin et al. designed an aeolotropic gel that was cross‐linked by metal nano‐components. This nanocomposite hydrogel possesses a highly ordered layered network structure.^64^ Due to the dynamic thiolate‐metal coordination, the nanocomposite hydrogel can respond to NIR and low pH conditions to achieve rapid and effective self‐healing properties. The hydrogel exhibits obvious anisotropy in mechanical, optical, and swelling behaviors, and thus it possesses controllable solvent responsiveness and mechanical driving ability. This anisotropic and self‐healing hydrogel exhibits huge application potential in the field of advanced soft bioactuators.

### 4.4 Recent advances in bioimaging nanocomposite hydrogels

Common imaging methods in the biomedical field include magnetic resonance (MRI), photoacoustic (PAI), and fluorescence imaging (FLI). Combining nanomaterials with imaging functions and hydrogels with good biocompatibility to obtain bioimaging nanocomposite hydrogels.^14^, ^160^ In composite systems, nanomaterials are generally bioimaging components that can respond to in vitro conditions (magnetic resonance, photoacoustic, fluorescence). While hydrogels can greatly improve the residence time of nanomaterials in target parts and avoid them being eliminated too quickly by the body. Bioimaging has the advantages of being noninvasive, functioning in real‐time, and allowing for dynamic monitoring, and this is extremely important for anti‐tumor therapies, tissue repair, and cell behavior monitoring.^161^

#### 4.4.1 Antitumor bioimaging

**FIGURE 10:**
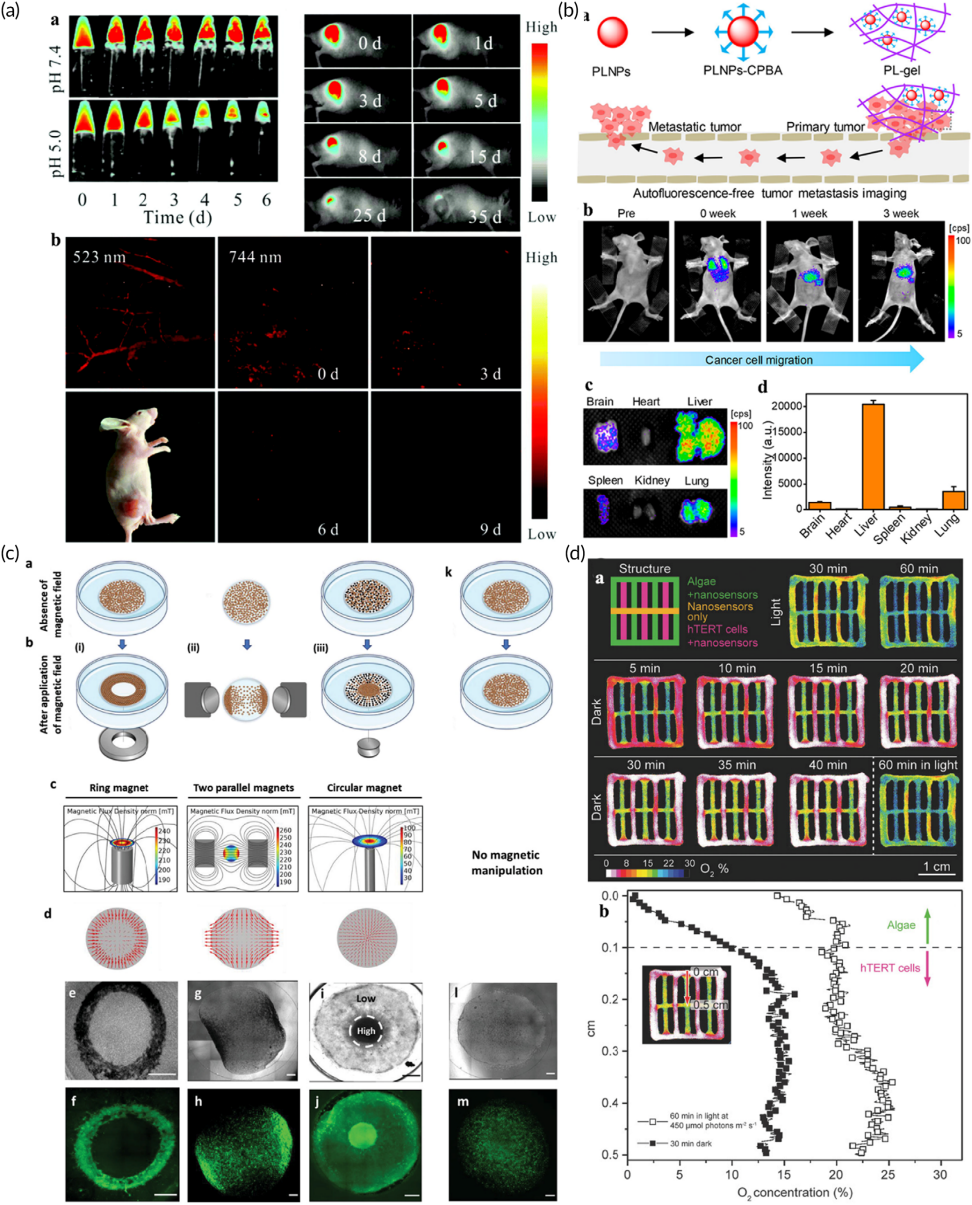
(A) (a) The degradation of hydrogel scaffolds in vitro and in vivo by FLI. (b) The degradation of hydrogel scaffolds in vivo by PAI. *Source*: Adapted with permission from ref 162. Copyright 2019, the Royal Society of Chemistry. (b) (a) The PLNP nanocomposite hydrogel targets and tracks tumor metastasis. (b) Sustained fluorescence images of tumor cells labeled with PLNPs‐CPBA to assess their tumor migration process. (c) Long‐lasting luminous image of mouse organs in vitro. (d) Semi‐quantitative analysis of continuous luminescence from different isolated organs. *Source*: Adapted with permission from ref 163. Copyright 2020, American Chemical Society. (c) Engineering design of 3D heart tissue operated through a magnetic field. (a, b) Diagram of the experimental procedure. (c, d) Magnetic field and force distribution of the different magnets. (e–j) The bright field and fluorescent images of the patterned tissue in response to different magnetic fields. (k–m) The control hydrogel without applying a magnetic field revealed the random distribution of CMs within the hydrogel. Scale bar: 1 mm. *Source*: Adapted with permission from ref 164. Copyright 2019, WILEY‐VCH. (d) (a) The images of O_2_ concentration distribution in the scaffold with or without light. (b) After 60 min of illumination or 30 min of darkness, the distribution of O_2_ concentration in the vertical direction of the scaffold was observed. *Source*: Adapted with permission from ref 165. Copyright 2018, WILEY‐VCH

The nanocomposite hydrogels used for anti‐tumor bioimaging are commonly biodegradable, and the degradation status in vivo can be monitored dynamically using biological imaging technology. Jin et al. designed a gel that consisted of coiled‐coil polypeptides, and the hydrogel contained Ag_2_S NPs and paclitaxel (PTX) that allow it to be used for chemo‐phototherapy of tumors (Figure 10a). The degradation of hydrogel was positively correlated with the release of Ag_2_S NPs. Therefore, the degradation of the hydrogel at tumor sites can be monitored dynamically through near‐infrared FLI and PAI of Ag_2_S.^162^ Except for degradation, the delivery process of nanomedicine in hydrogel systems can also be monitored through bioimaging technology. Dong et al. designed a gel platform loaded with DOX and cytosine‐phosphate‐guanine nanoparticles (CPG NPs) that can be used for chemo‐immunotherapy of tumors. According to the fluorescence characteristics of DOX and of CPG NPs, a dual FLI system was designed.^166^ The system reveals the entire dynamic process of chemical immunotherapy, including the stability and metabolic time of chemical drugs and adjuvants, the location and degradation of the gel system, and the movement of drugs. Additionally, malignant metastasis will occur when the tumor develops to a certain stage, and these metastatic events seriously threaten the lives of patients. Therefore, it is necessary to visualize the tumor metastasis process. Zhao et al. developed a nanocomposite hydrogel possessing long‐lasting luminescence by integrating tumor‐targeted nanoparticles (PL NPs) with alginate hydrogels (Figure 10b).^163^ PL NPs provide reproducible and long‐lasting NIR emission to achieve long‐term imaging and effectively avoid the interference of in situ radiation. The hydrogel can specifically mark tumors and track tumor metastasis.

#### 4.4.2 Tissue engineering bioimaging

Bioimaging possesses important potential in tissue bioimaging in vitro and dynamic monitoring of bone/cartilage regeneration in vivo. Limor Zwi‐Dantsis et al. designed functionalized magnetic nanoparticles (MNP) and combined them with a collagen hydrogel to develop a new platform that can be used to control tissue geometry (Figure 10C).^164^ MNP can target and label cardiomyocytes derived from pluripotent stem cells. Then, by imposing external magnetic fields to make the cells grow in a certain direction, the tissue can be given a specific shape without any mechanical support. At the same time, this process can be visualized in real‐time through bioimaging. Additionally, one of the advantages of bioimaging is that it can provide noninvasive monitoring. It is with great advantages in tissue engineering, particularly for in vivo tissues, such as bone and cartilage. Yang et al. modified USPIOs with kartogenin (KGN) and then combined them with cellulose nanocrystal/dextran hydrogel. KGN is an emerging non‐protein drug that can promote the differentiation of BMSCs into chondrocytes. USPIOs can achieve stable imaging in response to in vitro MRI.^97^ The nanocomposite hydrogel system could promote the regeneration of cartilage tissue, and it could also enable the noninvasive monitoring of the regenerated tissue through in vitro MRI. In addition, study shows that nanocomposite hydrogel can promote bone repair and achieve multimodal dynamic monitoring of bone repair process.^167^ These studies show that nanocomposite hydrogels have great potential in tissue engineering imaging.

#### 4.4.3 Cell behavior bioimaging

Nanocomposite hydrogels can be used to image living cells and active substances in the cells, which is useful for monitoring the physiological state of the cells and cell activities. Li et al. synthesized Ag NPs that could stably bound to the oligonucleotide and optimized the fluorescence characteristics of the Ag NPs.^168^ With the protection of DNA hydrogel, Ag NPs exhibited good light stability even in an oxygen environment, and they possessed a wide emission spectrum. Strong oxidant (ROS/RNS) decreased the red fluorescence and enhanced the green fluorescence of reductive Ag NPs. Thus, Ag NPs in DNA hydrogel can be used as fluorescence probes for cell imaging and for monitoring active oxygen species and nitrogen substances (ROS/RNS) in living cells, and this is of great significance for monitoring cell damage and cancer. Moreover, biological imaging technology can be used to dynamically monitor the metabolic type and chemical microenvironment of cells. Trampe et al. developed a functionalized biological ink by combining luminescent optical sensor nanoparticles with hydrogels, and this platform can be used for 3D bio‐printing of living cells and online imaging of O_2_ (Figure 10D).^165^ Optical sensor nanoparticles can perform noninvasive and online imaging of oxygen dynamics caused by cell respiration and photosynthesis. In this way, different types of metabolic activities in living cells can be distinguished in a completely 3D printed scaffold. This nanocomposite hydrogel bio‐ink can noninvasively draw the spatio‐temporal dynamics and chemical microenvironment of cell metabolism, thus allowing for rapid identification of cell activity in the 3D‐printed scaffold.

## 5 COMMERCIALIZATION OF NANOCOMPOSITE HYDROGELS IN BIOMEDICINE

In vivo applications include deep applications (tissue engineering, anti‐tumor, bioimaging), and superficial applications (transdermal MNs). Many limiting factors affect in vivo product transformation, the most important of which is the safety of the composite system.^169^ The product transformation of nanomaterials composite hydrogels is related to both hydrogels and nanomaterials. There are also several factors affect the safety of hydrogels, the most important of which are the matrix materials and the gelling methods. Commercialized matrix materials should reach the medical level to ensure safety. There are many gelling methods for hydrogels, but most of them used clinically are with bio‐orthogonal or non‐bio‐orthogonal gelling methods.^170^ Bio‐orthogonal can be performed in physiological environments, and the reaction conditions are generally mild, such as gelation based on ion interactions, hydrogen bonding and Schiff bases. Non‐bio‐orthogonal generally performed in harsh conditions, such as open‐loop polymerization caused by high temperature. Bio‐orthogonal reactions have important advantages in tissue engineering, while nonbio‐orthogonal reactions are clinically transformed relatively quickly.^170^ The safety of nanomaterials is mainly related to the material itself (such as element composition, nano size, surface charge). Among them, the size of nanomaterials is a factor affecting their biosafety and properties. For example, 10 nm Au NPs absorb green light and thus appear red. The melting temperature decreases dramatically as the size goes down. Moreover, 2–3 nm Au NPs are excellent catalysts, which also exhibit considerable magnetism. At this size, they are still metallic, but smaller ones turn into insulators. Their equilibrium structure changes to icosahedral symmetry, or they are even hollow or planar, depending on size.^171^ Therefore, in commercialization, it is also very important to select the appropriate size and ensure good size uniformity of nanomaterials. In addition, deep application in vivo usually requires higher safety than superficial application. The deeper the application depth of the product, the easier it is to contact the human blood circulation system and central nervous system, the higher the safety requirements of the product.^172^ Nanocomposite hydrogels also tend to be complex systems even when applied to shallow surfaces, which require high safety.

Biomedical products mainly contain medical devices and new drugs. Medical devices can be divided into Class I, II, and III according to the use risk of products from small to large.^173^ The new drugs require rigorous clinical trials, and the transformation is much more difficult. In medical devices, compared with hydrogels, the commercialization of nanomaterials is more difficult. For example, during the commercialization of medical devices, ISO 10993 is an international standard for evaluating their biocompatibility.^174^, ^175^ Standard published in 1992 (ISO 10993‐1:1992) is fully suitable for the safety evaluation of hydrogels, which promoted the development of hydrogel medical devices to a certain extent.^176^ The biocompatibility evaluation of hydrogel products is now relatively mature. However, as nanomaterials have different specific surface area and other factors, nanomaterials exhibit different biological properties from conventional materials. Until 2017, the international standard suitable for nanomaterials (ISO/TR10993‐22:2017) was released.^177^ The commercialization of nanomaterials is much later than that of hydrogels in medical devices. In addition, many countries have stricter approvals for nanomaterial medical devices. On FDA, the hydrogel‐based medical devices cover from Class I to Class III, while nanomaterial medical devices mainly cover from Class II to Class III. Moreover, there are much more records about *gel* (over 1000) than *nano* (only 200) on FDA (search “*gel*” or “*nano*” in 510(k) or PMA Database on FDA). In terms of new drugs, hydrogels and nanoparticles have many commercial products (Table 3). Hydrogels are mainly used as drug sustained‐release carriers. The great potential of nanomaterials in precision medicine promotes the commercialization of nanomaterials in new drugs. Currently, the nano drugs approved by FDA mainly include lipid‐based, protein‐based, polymeric and inorganic nanoparticles.^178^ Nano drugs are mainly used in the clinic to treat cancer, resolve mineral deficiencies, image tissues, and facilitate vaccination.^179^, ^180^, ^181^

**TABLE 3:** Representative products of nanomaterials or hydrogels

Products	Class	Application	Materials	Company
Restylane	Medical device (Class III)	Injectable dermal filler	Consists of hyaluronic acid (HA) hydrogel	Medicis Aesthetics Holdings, Inc
SpaceOAR Vue	Medical device (Class II)	Reduce the radiation dose delivered to the anterior rectum	Consists of absorbable polyethylene glycol (PEG)‐based hydrogel	Augmenix, Inc.
Zenieva	Medical device (Class I)	Wound dressing	Consists of crosslinked polyacrylic acid hydrogel	River's Edge Pharmaceuticals, LLC
NanoTite	Medical device (Class II)	Dental implants	Calcium phosphate nanoparticles	Implant Innovations, Inc.
NanoBone	Medical device (Class II)	Bone void filler	Nanocrystalline hydroxylapatite	ARTOSS GmbH
COBRA PzF	Medical device (Class III)	NanoCoated Coronary Stent	Polymeric polyphosphazene nanocoating	CeloNova BioSciences, Inc.
DEXTENZA	New drug	Treatment of eye inflammation	PEG‐based hydrogel contains dexamethasone	Ocular Therapeutix
VANTAS	New drug	Treatment of prostate cancer	Diffusion‐controlled hydrogel containing histrelin acetate	ENDO
SUPPRELIN LA	New drug	Children with central precocious puberty (CPP)	Hydrogel containing histrelin acetate	Indevus
RenaGel	New drug	Hyperphosphatemia	Hydrogel containing with sevalamer hydrochloride	GelTex Pharmaceuticals Inc.
DOXIL	New drug	Advanced ovarian cancer, multiple bone marrow cancer, and so on	Lipid‐based nanoparticles, liposomes (150–250 nm) loaded with DOX	Sequus Pharmaceuticals
mRNA‐1273	New drug	COVID‐19 vaccine	Lipid‐based nanoparticles, liposomes loaded with mRNA	Moderna
ADYNOVA‐TE	New drug	Hemophilia	Polymer‐based nanoparticles, PEGylated recombinant antihemophilia factor	Takeda
DexFerrum	New drug	Iron‐deficient anemia	Inorganic nanoparticles, iron dextran colloid	American regent
Abraxane	New drug	Advanced non‐small cell lung cancer Metastatic breast cancer (secondary) Metastatic pancreatic cancer (primary)	Protein‐based nanoparticles, albumin‐particle bound paclitaxel	Celgene
Feridex I.V.	New drug	Imaging of liver lesions	Inorganic nanoparticles, iron dextran colloid	AMAG

Nanocomposite hydrogels are complex systems composed of hydrogels and nanomaterials, and their commercialization in vivo is much more difficult. Biosafety evaluation of nanocomposite hydrogels is a much more complex process than that of nanomaterials or hydrogels. Until now, nanocomposite hydrogel products that can be used in vivo have not yet been approved by FDA, and only one clinical trial of nanomaterials composite hydrogels is found (ClinicalTrials.gov, Identifier: NCT04834245). This not only comes from the long cycle and high cost of R&D, production, and approval, but also comes from the difficulties in the production and material design of nanomaterials and hydrogels themselves.

Compared with in vivo applications, in vitro biomedical applications (biosensors, artificial skin, and biological actuators) pay more attention to the functionality and stability of the composite systems. This puts forward higher requirements for the material design and manufacture of nanomaterials and hydrogels. For in vitro applications, nanomaterials act more as the sensing core, endowing the composite system with electrical conductivity, being able to sense various small changes in the surrounding environment (temperature, stress, light, and substance concentration) and convert them into electrical signals.^57^, ^156^ Nanomaterials need to perform signal conversion repeatedly in this process, which puts forward higher requirements on the functionality and stability of nanomaterials. Therefore, in material designs, nanomaterials need to consider the good homogeneity, appropriate size, micromorphology, and surface chemical modification. In vitro applications also place extremely high requirements on the environmental tolerance of hydrogels. The hydrogels mainly determine the macroscopic phenotype of the composite systems. In vitro applications require hydrogels to withstand extreme temperatures, maintain humidity, wear resistance, strong flexibility, viscosity, degradation resistance, and appropriate swelling rate. In addition, the hydrogels must also have good retention of nanomaterials.^49^, ^57^ In designing, hydrogels often consider suitable polymer molecular weight, directional chemical modification, multicomponent combination, and so on. Manufacturing is also very important for product transformation. In the manufacturing process, it is necessary to control the cost of products, such as production capacity, input/output ratio, qualified product ratio, automation degree, and so on. At present, a trend is to expand the in vivo functions of in vitro products, such as integrating skin repair functions on biosensors.^153^ However, this puts forward higher safety requirements for the composite system and brings difficulties to the design and preparation of materials.

## 6 CONCLUSION AND OUTLOOK

Herein, we determined the composition and classification of nanomaterials and hydrogels. More importantly, we reviewed the recent advances of nanomaterials composite hydrogels in biomedicine, including emerging nanocomposite hydrogel MN technology, injectable nanocomposite hydrogels with minimally invasive advantages, self‐healing, and bioimaging nanocomposite hydrogels. The applications reviewed here encompass almost all areas of biomedicine, such as drug and cell delivery, cancer treatment, tissue regeneration, biosensing, and bioimaging. Many studies have shown that nanomaterials composite hydrogels possess high potential applicability in biomedicine. With the rapid development of 2DM, there is reason to believe that 2DM composite hydrogels will be the next big research hotspot. Although nanocomposite hydrogels have rich functions and most of them have good biocompatibility, their diffusion and metabolism in the body have not been definitively concluded. In addition, the possible off‐target toxicity and the long‐term introduction of physiological toxicity are still needed to be verified.

From in vivo to in vitro, nanocomposite hydrogels have infinite potential in biomedicine. However, product transformation faces resistance, especially in clinical transformation in vivo. The safety of composite systems is currently one of the main limiting factors. The transformation rate of in vitro products is relatively fast, but higher requirements are put forward for the stable functions and sustainability of the composite systems. Product transformation is extremely difficult, and it is necessary to gradually lower the threshold through interdisciplinary cooperation, especially from materials scientists, chemists, biologists, and clinicians. Looking forward to the future, nanomaterials composite hydrogels will shine in the fields of drug/cell delivery, tissue engineering, anti‐tumor, biosensing, bioimaging, and so on.

### PEER REVIEW

The peer review history for this article is available at https://publons.com/publon/10.1002/btm2.10315.

## Data Availability

Data sharing not applicable to this article as no datasets were generated or analysed during the current study.
